# Hypoxia induces mitochondrial protein lactylation to limit oxidative phosphorylation

**DOI:** 10.1038/s41422-023-00864-6

**Published:** 2024-01-02

**Authors:** Yunzi Mao, Jiaojiao Zhang, Qian Zhou, Xiadi He, Zhifang Zheng, Yun Wei, Kaiqiang Zhou, Yan Lin, Haowen Yu, Haihui Zhang, Yineng Zhou, Pengcheng Lin, Baixing Wu, Yiyuan Yuan, Jianyuan Zhao, Wei Xu, Shimin Zhao

**Affiliations:** 1 The Obstetrics & Gynecology Hospital of Fudan University, Shanghai Key Laboratory of Metabolic Remodeling and Health, State Key Laboratory of Genetic Engineering, School of Life Sciences, Children’s Hospital of Fudan University, and Institutes of Biomedical Sciences, Fudan University, Shanghai, China; 2 Department of Cancer Biology, Dana-Farber Cancer Institute, Boston, MA USA; 3 Department of Biological Chemistry and Molecular Pharmacology, Harvard Medical School, Boston, MA USA; 4 NHC Key Lab of Reproduction Regulation (Shanghai Institute of Planned Parenthood Research), Shanghai, China; 5 Shanghai Fifth People’s Hospital of Fudan University, Fudan University, Shanghai, China; 6 Key Laboratory for Tibet Plateau Phytochemistry of Qinghai Province, College of Pharmacy, Qinghai University for Nationalities, Xining, Qinghai China; 7 Guangdong Provincial Key Laboratory of Malignant Tumor Epigenetics and Gene Regulation, Guangdong-Hong Kong Joint Laboratory for RNA Medicine, RNA Biomedical Institute, Medical Research Center, Sun Yat-Sen Memorial Hospital, Sun Yat-Sen University, Guangzhou, Guangdong China

**Keywords:** Cell biology, Molecular biology

## Abstract

Oxidative phosphorylation (OXPHOS) consumes oxygen to produce ATP. However, the mechanism that balances OXPHOS activity and intracellular oxygen availability remains elusive. Here, we report that mitochondrial protein lactylation is induced by intracellular hypoxia to constrain OXPHOS. We show that mitochondrial alanyl-tRNA synthetase (AARS2) is a protein lysine lactyltransferase, whose proteasomal degradation is enhanced by proline 377 hydroxylation catalyzed by the oxygen-sensing hydroxylase PHD2. Hypoxia induces AARS2 accumulation to lactylate PDHA1 lysine 336 in the pyruvate dehydrogenase complex and carnitine palmitoyltransferase 2 (CPT2) lysine 457/8, inactivating both enzymes and inhibiting OXPHOS by limiting acetyl-CoA influx from pyruvate and fatty acid oxidation, respectively. PDHA1 and CPT2 lactylation can be reversed by SIRT3 to activate OXPHOS. In mouse muscle cells, lactylation is induced by lactate oxidation-induced intracellular hypoxia during exercise to constrain high-intensity endurance running exhaustion time, which can be increased or decreased by decreasing or increasing lactylation levels, respectively. Our results reveal that mitochondrial protein lactylation integrates intracellular hypoxia and lactate signals to regulate OXPHOS.

## Introduction

Oxidative phosphorylation (OXPHOS) is the major ATP-producing process that requires oxygen. Muscle cells upregulate OXPHOS to increase energy supply during exercise.^1^ This is achieved partly by switching to lactate oxidation via OXPHOS.^2^ Although these processes are consistent with the increase in ATP supply distributed via the mitochondrial reticulum for muscle contraction during exercise,^3^ it raises a question regarding how muscle cells maintain a balance between oxygen-consuming OXPHOS that generates ATP and the hypoxic conditions created by exercise. This is because, although the intramuscular oxygen supply is increased by enhanced pulmonary diffusion capacity, cardiac output, the oxygen-carrying capacity of the blood, and skeletal muscle oxygen extraction during excercise,^4^ the hypoxia, i.e., the decrease of partial pressure of oxygen (PO_2_) from ∼40 Torr, at rest, to 3–4 Torr under exercise at about 65% of maximal oxygen consumption (VO_2_max),^1,2^ is induced in muscles. Moreover, the mechanism utilized by muscle cells to prevent the overproduction of reactive oxygen species (ROS), which occurs under endurance exercise-associated hypoxia^5,6^ and leads to oxidative stress in cells and tissues, remains unclear.

Cellular physiology adapts to oxygen availability by sensing oxygen availability via the prolyl hydroxylases (PHDs)-von Hippel-Lindau (VHL)^7^-hypoxia-inducible factor α (HIFα)^8^ axis. PHDs use oxygen and α-ketoglutarate as substrates to hydroxylate proline residues in proteins containing PHD-recognizing sequences.^9^ Hydroxylated proteins, such as HIF1α, are subsequently recognized and degraded by VHL, an E3 ligase.^10^ HIFαs are transcription factors that regulate an array of processes, such as glucose uptake, glycolysis,^11^ and vascularization^12^ that provide substrates for OXPHOS. However, HIFαs do not directly regulate muscle cell OXPHOS, but only exert transcriptional regulation, which takes hours to be effective, and thus lag behind exercise-induced changes in OXPHOS in muscles that take only minutes to manifest after starting to exercise.

Lactate is quickly induced by exercise or heartbeat, due to enhanced aerobic glycolysis and glycogen catabolism that elevates pyruvate,^13^ which is then thermodynamic favorably converted to lactate by lactate dehydrogenase A (LDHA).^1^ Besides LDHA, lactate homeostasis is also determined by the pyruvate dehydrogenase complex (PDC) that channels pyruvate to acetyl-CoA (Ac-CoA) as an OXPHOS substrate. In muscle cells, the PDC, which is activated during exercise due to changes taking place in the NADH/NAD^+^ ratio and metal ions,^14–17^ can be inactivated by acetylation,^18^ and by PHD3 binding to PDC E1 beta (PDHE1B),^19^ both leading to an increase in phosphorylation of serines 232, 293, and 300 of PDHA1,^20,21^ the catalytic subunit of PDC. Limiting Ac-CoA, originating from glucose, glycogen, and lactate^22,23^ catabolism via PDC^1^ or from fatty acid oxidation (FAO), inhibits OXPHOS and reduces oxygen consumption. However, lactate, while serving as a preferred fuel source for muscle and heart cells via OXPHOS, inhibits lipolysis^24,25^ and carnitine palmitoyltransferase 1 (CPT1)-mediated fatty acid import into mitochondria,^26^ showing that lactate exerts both positive and negative regulation of OXPHOS.

Mechanisms underlying the sensing of lactate as well as the generation of lactate signals to regulate OXPHOS remain unclear. Lactate signaling is highlighted by the modification of lysine in proteins by lactate,^27,28^ although a bona fide lactyltransferase remains elusive. Mutations in mitochondrial alanyl‐tRNA synthetase 2 (AARS2),^29–31^ which catalyzes alanyl‐tRNA formation in mitochondrial protein synthesis^32,33^ and induces lysine alanylation in proteins,^27,28,34,35^ cause impaired OXPHOS, lactic acid deregulation and muscle function loss, with underlying mechanisms undefined.^29–31,36^ We were intrigued by the possibility that AARS2 might act as a potential lactyltransferase that regulates muscle OXPHOS. In this study, we aimed to determine whether AARS2 acts as a mitochondrial lactyltransferase that integrates intracellular hypoxia and lactate signals to regulate muscle cell OXPHOS.

## Results

### AARS2 contains PHD-recognizing sequence and is hydroxylated by PHD2

**Fig. 1:**
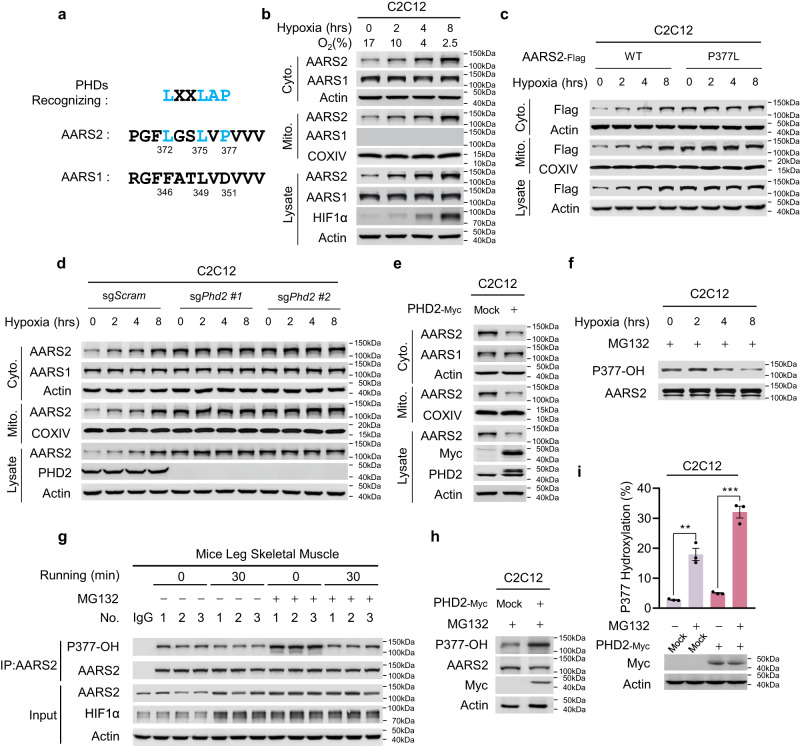
Oxygen regulates AARS2 protein levels via PHD2. **a** AARS2 carries a PHD-recognizing sequence. The amino acid (aa) sequence from 369 to 380 of AARS2 and its corresponding AARS1 sequence (aa 343–354) were aligned to the PHD-recognizing sequence. Consensus PHD-recognizing residues are marked in blue. **b** AARS2 protein levels are regulated by hypoxia. Mitochondrial and cytosolic AARS1, AARS2 and HIF1α levels were detected in C2C12 cells cultured in a hypoxia chamber for the indicated time durations. COXIV and actin were used to demonstrate the successful isolation of mitochondria and cytoplasm, respectively (the same method was employed for subcellular fractionation henceforth). Chamber oxygen levels were monitored. **c** P377 is required for the regulation of AARS2 protein levels by hypoxia. Stably expressed AARS2^WT^ and AARS2^P377L^ protein (P377L) levels in C2C12 cells were measured after the cells were cultured in a hypoxia chamber for the indicated time durations. **d**, **e** PHD2 regulates AARS2 protein levels. Mitochondrial and cytosolic AARS1 and AARS2 levels were measured in C2C12 cells or *Phd2* KO C2C12 cells cultured in a hypoxia chamber for the indicated time durations (**d**) and in C2C12 cells overexpressing PHD2 (**e**). **f**–**i** Hypoxia and PHD2 regulate P377 hydroxylation. P377OH levels were determined via western blot assay in C2C12 cells cultured in a hypoxia chamber for indicated time durations in the presence of MG132 in the culture media to prevent proteasomal degradation (**f**); in the resting and 30 min running mouse leg skeletal muscles that were intravenously injected with or without 1 mg/kg MG132 twice a week for 4 consecutive weeks (**g**) (*n* = 3); in the PHD2 overexpressing C2C12 cells treated with MG132 (**h**). P377OH levels in PHD2 overexpressing C2C12 cells in the presence and absence of MG132 were quantified by mass spectrometry (**i**) (*n* = 3). All data are reported as mean ± SEM of three independent experiments. Statistical significance was assessed by two-way ANOVA: **P < 0.01; ***P < 0.001.

AARS2 proline 377 (P377) lies in a PHD-recognizing sequence,^9^ which is not present in the corresponding sequence of cytosolic alanyl-tRNA synthetase 1 (AARS1) (Fig. 1a), indicating that P377 may be hydroxylated, allowing AARS2 levels similar to HIF1α,^9,37^ to be regulated during hypoxia. This was confirmed by the observation that, although hypoxia time-dependently induced AARS2 expression in both mitochondria and the cytoplasm of mouse proliferating myoblasts C2C12 cells, mouse HL-1 cardiomyocytes, and primary mouse hepatocytes, AARS1 was expressed only in the cytosol and was not induced by hypoxia (Fig. 1b; Supplementary information, Fig. S1a, b). Hypoxia also induced AARS2 protein levels in mouse primary myoblasts over time (Supplementary information, Fig. S1c). Moreover, AARS2 protein levels did not change with hypoxia when P377 was switched to leucine (Fig. 1c), which substantiated the observation that hypoxia-mediated upregulation of AARS2 protein levels requires P377. Silencing or knocking out *Phd2*, but not other oxygen-sensing proline hydroxylases, such as *Phd1* and *Phd3*, in C2C12 cells elevated the levels of endogenous AARS2 (Supplementary information, Fig. S1d) and prevented hypoxia-mediated accumulation of endogenous AARS2 (Fig. 1d). These results, together with the observation that PHD2 overexpression decreased AARS2 levels in C2C12 cells (Fig. 1e), whereas inhibiting PHDs with roxadustat increased them (Supplementary information, Fig. S1e), confirmed that PHD2 acts as a proline hydroxylase and oxygen sensor for AARS2.

We searched for hydroxylated P377 (P377OH)-containing peptides in the 120 tandem mass (MS/MS) spectra of a Glu-C protease peptide library (Supplementary information, Table S1) of AARS2 overexpressing HEK293T cells, and found 3 spectra matching the projected MS/MS spectrum of a P377OH-containing AARS2 peptide (Supplementary information, Fig. S1f). As a synthetic P377OH-containing Glu-C AARS2 peptide generated a spectrum identical to that of the identified MS/MS spectrum (Supplementary information, Fig. S1f), we concluded that P377 was hydroxylated in vivo. Therefore, to detect P377OH in cells, we generated a P377OH site-specific antibody that specifically recognizes P377OH in the peptide of intact AARS2 (Supplementary information, Fig. S1g, h). The P377OH levels of ectopically expressed AARS2 in C2C12 cells and mouse primary myoblasts were time-dependently decreased by hypoxia exposure in the presence of MG132, a proteasome inhibitor (Fig. 1f; Supplementary information, Fig. S1i). P377OH levels decreased in mouse leg muscles in which protein degradation was inhibited by MG132 (Fig. 1g) after hypoxia was induced by running (Supplementary information, Fig. S1j), confirming that P377OH is regulated by oxygen availability physiologically. Additionally, PHD2 overexpression increased P377OH levels in C2C12 cells, further to which, both MG132 and PHD2 enhanced the cellular P377OH levels synergistically (Fig. 1h, i). This result confirmed that P377OH, which possibly acts as a negative regulator of AARS2, is in turn modulated by PHD2.

### AARS2 acts as a substrate of the PHD2–VHL proteasomal machinery

**Fig. 2:**
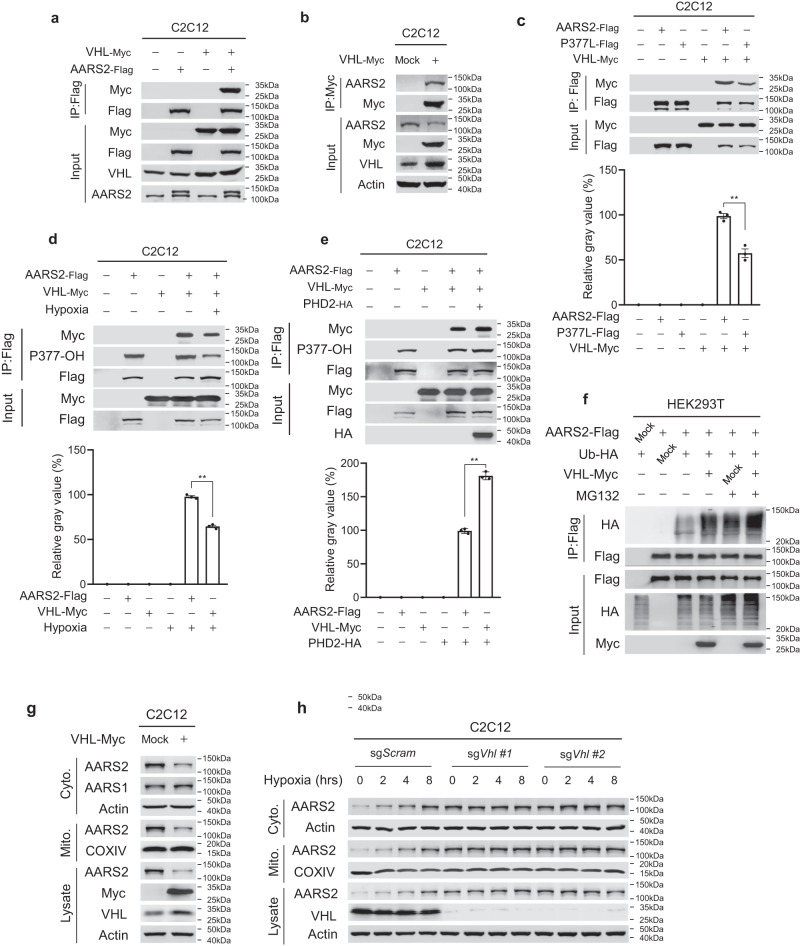
AARS2 acts as a substrate of the PHD2–VHL proteasomal machinery. **a**, **b** VHL interacts with AARS2. Co-immunoprecipitated, co-expressed (**a**) and endogenous (**b**) AARS2 was detected in C2C12 cells ectopically expressing VHL. **c** P377L mutant shows a weakened affinity for VHL. Interactions between VHL and AARS2, and between VHL and P377L were detected via co-immunoprecipitation when they were co-expressed in C2C12 cells. Densitometric analysis for images was provided. **d**, **e** VHL–AARS2 interaction is regulated by hypoxia and PHD2. The amount of AARS2 co-purified VHL was determined under normoxia and 8 h hypoxia (**d**), as well as with or without PHD2 overexpressing (**e**) in C2C12 cells. Densitometric analysis for images was provided. **f** VHL mediates proteasomal degradation. AARS2 ectopically expressed in HEK293T cells was assayed for ubiquitination when expressed alone and when co-expressed with ubiquitin or VHL, or both, under the presence and absence of MG132. **g** VHL downregulates AARS2 protein levels. Mitochondrial and cytosolic AARS2 levels were measured in C2C12 cells with or without VHL overexpression. **h** Hypoxia induces AARS2 accumulation in a VHL-dependent manner. Mitochondrial and cytosolic AARS2 levels were measured in C2C12 cells and *Vhl* KO C2C12 cells both cultured in a hypoxia chamber for the indicated time durations. All data are reported as mean ± SEM of three independent experiments. Statistical significance was assessed by unpaired two-tailed Student’s *t*-test: **P < 0.01.

Ectopically expressed VHL co-precipitated with overexpressed and endogenous AARS2 in C2C12 cells (Fig. 2a, b), consistent with endogenous AARS2 interacting with endogenous PHD2 and VHL in mouse primary myoblasts (Supplementary information, Fig. S1k), suggesting that AARS2 is a substrate of VHL. When co-expressed in C2C12 cells, the interaction between wild-type AARS2 and VHL was greater than that between AARS2 harboring P377L and VHL (Fig. 2c); moreover, the AARS2–VHL interaction was decreased and increased by hypoxia (Fig. 2d) and PHD2 overexpression (Fig. 2e), respectively, indicating that P377OH increased the VHL–AARS2 interaction. VHL overexpression increased the ubiquitination levels of AARS2 expressed in HEK293T cells (Fig. 2f), whereas it reduced endogenous mitochondrial and cytosolic AARS2 protein levels in C2C12 cells (Fig. 2g). Conversely, *Vhl* knockout (KO) in C2C12 cells increased endogenous AARS2 levels and abrogated the hypoxia-mediated increase in AARS2 levels (Fig. 2h). These results collectively confirmed that VHL degrades AARS2.

### AARS2 inhibits Ac-CoA production and OXPHOS

**Fig. 3:**
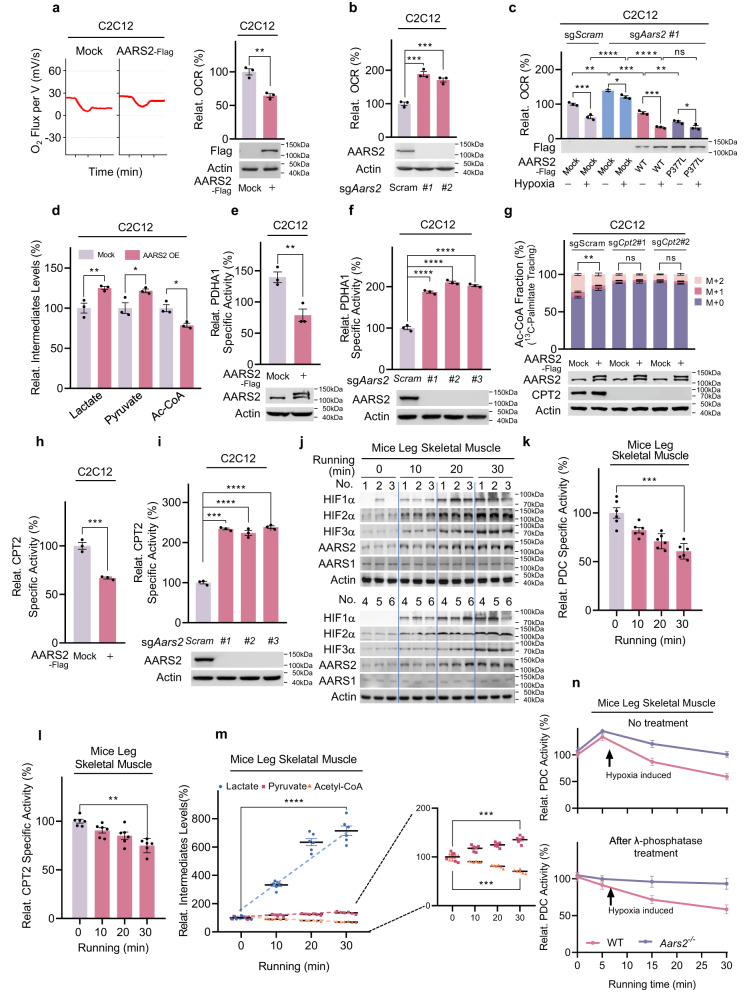
AARS2 inhibits Ac-CoA production and OXOPHOS. **a**, **b** AARS2 regulates OCR. The effects of overexpressing AARS2 (left, OROBOROS Oxygraph-2K measurements; right, quantitation) (**a**), and *Aars2* KO using independent sgRNAs (**b**) on OCR (O_2_ influx per volume cells, mV/s) were determined (*n* = 3). **c** AARS2 regulates OCR in response to oxygen levels. The effects of re-introducing wide-type AARS2 or P377L into *Aars2*^*−/−*^ C2C12 cells were determined in cells under normoxic conditions and cells which were exposed to hypoxia for 8 h (*n* = 3). **d** AARS2 regulates metabolite levels. Relative levels (to those of C2C12 cells) of lactate, pyruvate, and Ac-CoA in C2C12 cells and C2C12 cells overexpressing AARS2 (*n* = 3) were evaluated. **e**, **f** AARS2 regulates PDHA1-specific activity. Relative specific activities (to those from C2C12 cells) of PDHA1 purified from C2C12 cells overexpressing AARS2 (**e**) and *Aars2* KO C2C12 cells (**f**) were determined (*n* = 3). **g** AARS2 regulates CPT2-mediated Ac-CoA production. The percentages of unlabeled (M + 0), single labeled (M + 1), and double-labeled (M + 2) Ac-CoA from ^13^C-palmitate in C2C12 cells and C2C12 cells in which *Cpt2* had been knocked out using independent sgRNAs, were determined with or without AARS2 overexpression; 100 μM ^13^C-palmitate chasing was performed for 12 h (*n* = 3). The M + 2 percentages in C2C12 cells with and without AARS2 overexpression were compared for significance. **h**, **i** AARS2 regulates CPT2-specific activity. The relative specific activity (to those from C2C12 cells) of CPT2, isolated from both AARS2-overexpressing (**h**) and *Aars2* KO C2C12 cells (**i**) was determined (*n* = 3). **j** Running induces HIFαs and AARS2 expression in mouse leg skeletal muscles. HIF1α, HIF2α, HIF3α, AARS1, and AARS2 levels in mouse leg skeletal muscles were determined before and after running for the indicated durations (*n* = 6). **k**, **l** Running inactivates PDC and CPT2 in vivo. The relative (to those of resting mice) specific activities of PDC (**k**) and CPT2 (**l**) in mouse leg skeletal muscles were determined after mice started running for indicated durations (*n* = 6). **m** Endurance running-induced lactate accumulation and Ac-CoA reduction. The lactate, pyruvate, and Ac-CoA levels in mouse leg skeletal muscles were determined after mice were allowed to run for the indicated durations (*n* = 6). **n** AARS2 inactivates PDC via a mechanism other than phosphorylation. The relative PDC activities (to protein amount) of wild-type and *Aars2*^*−/−*^ mouse leg skeletal muscles sampled at different time points after starting to run with or without dephosphorylation induced by λ phosphatase, were determined (*n* = 6). The activities at time 0 were set as 100%. All data are reported as mean ± SEM of three independent experiments. Statistical significance was assessed by unpaired two-tailed Student’s *t*-test and two-way ANOVA: *P < 0.05; **P < 0.01; ***P < 0.001; ****P < 0.0001; ns no significance.

AARS2 overexpression decreased the oxygen consumption rate (OCR) in C2C12 cells (Fig. 3a). *Aars2* KO (*Aars2*^*−/−*^) increased OCR and diminished OCR response to hypoxia in C2C12 cells, and these consequences were rescued by reintroducing wild-type AARS2, but not the AARS2 P377L mutant into *Aars2*^*−/−*^ cells (Fig. 3b, c), indicating that AARS2 responded to hypoxia by inhibiting cellular respiration. Additionally, AARS2 overexpression decreased Ac-CoA levels but increased pyruvate and lactate levels in C2C12 cells (Fig. 3d), suggesting that AARS2 negatively regulated PDC, the enzyme complex that converts pyruvate to Ac-CoA. This was further supported by the observation that ectopically overexpressing AARS2 to double cellular AARS2 levels decreased the specific activity of PDHA1 (Fig. 3e), whereas *Aars2* KO increased it (Fig. 3f). PDHA1 activities increased while their responses to hypoxia were abrogated in *Aars2* KO C2C12 cells; the responses to hypoxia were however, restored by reintroducing wild-type, but not P377L mutant AARS2 (Supplementary information, Fig. S2a). Moreover, AARS2 overexpression did not alter the phosphorylation levels of serines 232, 293, and 300 of PDHA1 (Supplementary information, Fig. S2b), which are known to inactivate PDHA1, implying that AARS2 inactivates PDHA1 via a novel mechanism under the control of hypoxia and AARS2.

As CPT1/2/FAO inhibition may also contribute to OCR inhibition, by decreasing Ac-CoA supply to OXPHOS, ^13^C-palmitate was used to investigate whether CPT1/2/FAO was inhibited by AARS2 (Supplementary information, Fig. S2c). AARS2 overexpression decreased double-labeled (M2) ^13^C-Ac-CoA production from ^13^C-palmitate (Fig. 3g), indicating that AARS2 inhibited CPT1/2/FAO. Only endogenous carnitine palmitoyltransferase 2 (CPT2), and not CPT1 or FAO enzymes, interacted with ectopically expressed AARS2 (Supplementary information, Fig. S2d). Moreover, AARS2 overexpression decreased CPT2 activity (Fig. 3h), whereas *Aars2* KO increased it (Fig. 3i), confirming that AARS2 inactivates CPT2 possibly via a covalent modification. Furthermore, *Aars2* KO activated CPT2 and diminished responses of CPT2 activity to hypoxia, which were rescued by reintroducing wild-type AARS2, but not AARS2 P377L mutant (Supplementary information, Fig. S2e). *Cpt2* KO in C2C12 cells rendered ^13^C-Ac-CoA production unresponsive to AARS2 overexpression (Fig. 3g). Finally, neither CPT1 specific activity (Supplementary information, Fig. S2f) nor protein level (Supplementary information, Fig. S2g) was affected by AARS2 overexpression. These data collectively suggested that AARS2 inactivates CPT2 to inhibit FAO.

We used ^13^C-labeled glutamine to trace α-ketoglutarate (α-KG) influx, which is the point at which amino acids feed into the TCA cycle (Supplementary information, Fig. S2h). The ^13^C-α-KG levels were not affected by AARS2 overexpression (Supplementary information, Fig. S2i), suggesting that amino acid catabolism was not regulated by AARS2. These results collectively suggested that AARS2 inhibits respiration by decreasing Ac-CoA supply from glycolysis and FAO by inactivating PDHA1 and CPT2, and not via amino acid catabolism (Supplementary information, Fig. S2j).

Next, we verified whether hypoxia mediates PDHA1 and CPT2 regulation via AARS2 in vivo. HIFαs and AARS2 protein levels were induced in mouse leg skeletal muscles in a time-dependent manner while mice were running on a treadmill (Fig. 3j). Moreover, muscle Ac-CoA level, which was moderately decreased while muscle PDC- (Fig. 3k) and CPT2- (Fig. 3l) specific activities kept decreasing, and hypoxia in leg muscle (Supplementary information, Fig. S3a) as well as lactate and pyruvate levels kept increasing, after running started (Fig. 3m). This was coincident with a brief increase in the total PDC activity in mouse leg skeletal muscles, which was then followed by a decrease to levels below the resting levels (Fig. 3n). The initial increase in total PDC activity was consistent with an initial decrease in PDHA1 phosphorylation (Supplementary information, Fig. S3b) and λ phosphatase treatment to diminish phosphorylation effects (Supplementary information, Fig. S3c) abrogated initial increase (Fig. 3n). However, the decrease in PDC activity with running time was unaffected even after λ phosphatase treatment (Fig. 3n). These, together with the observation that neither the protein levels of PDC components and CPT2 (Supplementary information, Fig. S3d) nor the protein levels and activities of LDHs (Supplementary information, Fig. S3d, e) were affected throughout 30 min running, suggesting that AARS2 may mediate a covalent modification mechanism which inactivates PDC without involving PDHA1 phosphorylation.

### Lactate inactivates PDHA1 and CPT2 dependent on AARS2

**Fig. 4:**
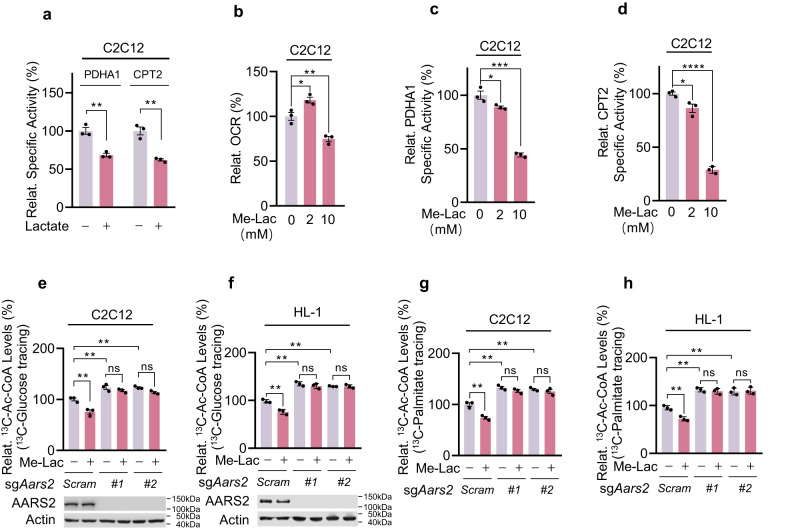
Lactate inactivates PDHA1 and CPT2 dependent on AARS2. **a** Lactate treatments inactivate cellular PDHA1 and CPT2. C2C12 cells were treated with 10 mM lactate. The relative (to those from untreated C2C12 cells) specific activities of PDHA1 and CPT2 were determined (*n* = 3). **b** Me-Lac treatments decrease cellular OCRs. OCRs of C2C12 cells that were untreated or treated with 2 mM or 10 mM Me-Lac for 4 h (*n* = 3) were determined. **c**, **d** Me-Lac treatments inactivate cellular PDHA1 and CPT2. Relative specific activities (to those of untreated C2C12 cells) of PDHA1 (**c**) and CPT2 (**d**) in C2C12 cells that were untreated or treated with 2 mM or 10 mM Me-Lac (*n* = 3) were determined. **e**–**h** Lactate treatments decrease Ac-CoA influx from glycolysis and FAO. Relative ^13^C-Ac-CoA levels in ^13^C-glucose (**e**, **f**) and ^13^C-palmitate (**g**, **h**) (to untreated C2C12 cells) and ^13^C-Ac-CoA (M + 2 only)-treated C2C12 cells, *Aars2* KO C2C12 cells (**e**, **g**) and HL-1 cells, *Aars2* KO HL-1 cells (**f**, **h**) were detected before and after being treated with 10 mM Me-Lac (*n* = 3). The chasing time for 10 mM ^13^C-glucose and 100 μM ^13^C-palmitate was 1 h and 12 h, respectively. All data are reported as mean ± SEM of three independent experiments. Statistical significance was assessed by unpaired two-tailed Student’s *t*-test and two-way ANOVA: *P < 0.05; **P < 0.01; ***P < 0.001; ****P < 0.0001; ns no significance.

Lactate decreased PDHA1 and CPT2-specific activities (Fig. 4a). Cell membrane permeable methyl-lactate (Me-Lac) was employed to analyze lactate effects. Increased OCRs, as well as decreased PDHA1 and CPT2-specific activities, were observed in C2C12 cells treated with 2 mM Me-Lac, which increased the mitochondrial lactic acid levels from 0.6 mM to 1.0 mM; however, Me-Lac at 10 mM, which increased mitochondrial lactic acid to 3.2 mM, decreased OCRs, as well as the specific activities of PDHA1 and CPT2 (Fig. 4b–d; Supplementary information, Fig. S4a). The 2 mM Me-Lac treatment results were consistent with the observation that lactate fuels muscle cells via the TCA cycle.^1^ However, the 10 mM Me-Lac treatment, results in intracellular lactate levels mimicking those in muscle during exercise, suggesting that lactate also plays inhibitory roles in OXPHOS, likely via a covalent modification to PDHA1 and CPT2, a notion confirmed by Me-lac decreasing PDHA1 and CPT2 activity in C2C12 cells, but not in *Aars2* KO C2C12 cells (Supplementary information, Fig. S4b, c). The above notion was further confirmed by examining Ac-CoA production in lactate-oxidizing C2C12 and HL-1 cells, which revealed that 10 mM Me-Lac decreased ^13^C-Ac-CoA production from ^13^C-glucose (Fig. 4e, f) and ^13^C-palmitate (Fig. 4g, h) in an AARS2-dependent manner. Intriguingly, loss of AARS2 increased ^13^C-Ac-CoA production from^13^C-glucose and ^13^C-palmitate (Fig. 4e–h), indicating that lactate inactivated PDHA1 and CPT2 via AARS2, and that lactate does not inactivate PDHA1 and CPT2 and OXPHOS via its acidity. Moreover, 10 mM Me-Lac inhibited OCR in primary myoblasts in an AARS2-dependent manner (Supplementary information, Fig. S4d). Further, Me-Lac negligibly affected ^13^C-α-KG production from ^13^C-glutamine (Supplementary information, Fig. S4e), which was consistent with that amino acid catabolism was not regulated by lactic acid.

### AARS2 inactivates PDHA1 and CPT2 by lactylating these proteins

**Fig. 5:**
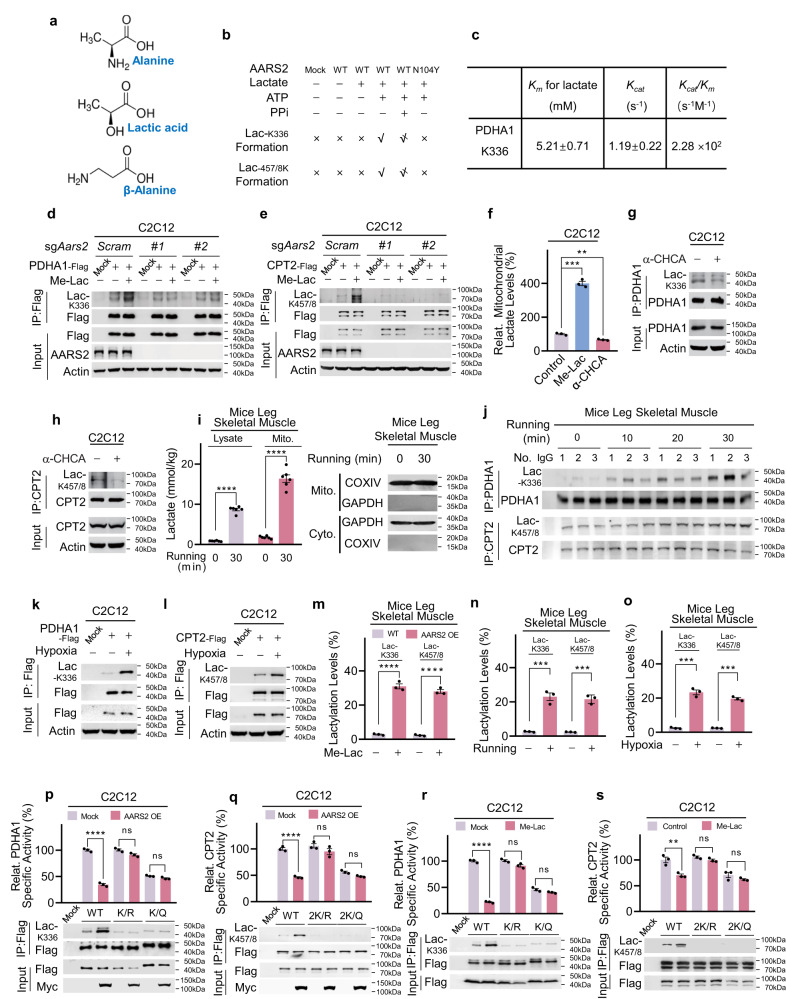
AARS2 inactivates PDHA1 and CPT2 by lactylating these proteins. **a** Lactic acid, β-alanine, and alanine are analogs. Alignment of chemical structures of alanine, lactic acid, and β-alanine. **b** AARS2 lactylates PDHA1 K336 and CPT2 K457/8 peptides in vitro. MS-based detection of lactylated synthetic PDHA1 K336 and CPT2 K457/8 peptides catalyzed by AARS2 and AARS2^N104Y^ are summarized (×: no product detected, √: product detected, : less product detected; see Supplementary information, Fig. S6b for MS results). **c** AARS2 is an efficient lactyltransferase. The *K*_*m*_ and *K*_*cat*_ of recombinant AARS2 toward lactate were determined when the synthetic K336-containing PDHA1 peptide was kept at 0.5 mg/mL (*n* = 3). **d**, **e** *AARS2* KO abolishes lactate-linked induction of Lac-K336 and Lac-K457/8. Lac-K336 levels (**d**) and Lac-K457/8 levels (**e**) were detected for PDHA1 and CPT2 purified from C2C12 cells and C2C12 cells in which *Aars2* had been knocked out using independent sgRNAs, with or without 10 mM Me-Lac in the culture media. **f**–**h** Inhibiting the import of lactate into mitochondria decreases Lac-K336 and Lac-K457/8 levels. C2C12 cells that were either untreated, treated with 5 mM α-CHCA for 2 h, or with 10 mM lactate for 4 h. Mitochondrial lactate (**f**) (*n* = 3), Lac-K336 (**g**) and Lac-K457/8 (**h**) were detected. **i** Running proportionally increases lactate levels in mitochondria. Mitochondrial and lysate lactate levels in mouse leg skeletal muscles were detected before and after 30 min of running (*n* = 6). Success mitochondria extraction isolation was confirmed by staining both mitochondria marker COXIV and cytosolic marker GAPDH (here in after). **j** Running induces Lac-K336 and Lac-K457/8. Lac-K336 and Lac-K457/8 levels in purified PDHA1 and CPT2 of mouse leg skeletal muscles were detected after running for indicated time durations (*n* = 3). **k**, **l** Hypoxia-induced Lac-K336 and Lac-K457/8. Lac-K336 (**k**) and Lac-K457/8 (**l**) levels of PDHA1 and CPT2 in C2C12 cells were detected before and after they were exposed to hypoxia for 8 h. **m** Combined AARS2 and lactate treatments increased Lac-K336 and Lac-K457/8 levels. Lac-K336 and Lac-K457/8 levels in the leg skeletal muscles of resting mice and AARS2-overexpressing mice given lactate gastrocnemius muscle injections were estimated using MS (*n* = 3). **n**, **o** Running and hypoxia increased Lac-K336 and Lac-K457/8 levels. Lac-K336 and Lac-K457/8 levels in the mouse leg skeletal muscles before and after 30 min of running (**n**) and perfused with Krebs Ringer solution aerated with or without O_2_ in a 1200 A Isolated Intact Muscle Test System for 30 min (**o**) were estimated using MS (*n* = 3). **p**, **q** AARS2 inactivates PDHA1 and CPT2 by inducing lactylation. The effects of AARS2 overexpression on lactylation levels and specific activities (*n* = 3) of PDHA1 (**p**) and CPT2 (**q**) and their respective lactylation site mutants were detected. **r**, **s** Lactate inactivates PDHA1 and CPT2 by inducing lactylation. The effects of 10 mM Me-Lac on Lac-K336 and the specific activities of PDHA1 and its lactylation site mutants (**r**), and the effects of Me-Lac on Lac-K457/8 and the specific activities of CPT2 and its lactylation site mutants (**s**) were detected (*n* = 3). All data are reported as mean ± SEM of three independent experiments. Statistical significance was assessed by unpaired two-tailed Student’s *t*-test and two-way ANOVA: **P < 0.01; ***P < 0.001; ****P < 0.0001; ns no significance.

Lactic acid is structurally analogous to alanine (Fig. 5a). The binding of lactic acid to AARS2 was modeled using molecule docking analysis (Supplementary information, Fig. S4f) and confirmed via isothermal titration calorimetry (ITC) (Supplementary information, Fig. S4g). Moreover, overexpressed AARS2 interacted with both endogenous PDHA1 (Supplementary information, Fig. S4h) and ectopically expressed CPT2 (Supplementary information, Fig. S4i). These results collectively suggested that AARS2 may function as a lactyltransferase for PDHA1 and CPT2. To confirm this hypothesis, we first searched for lactylated lysine residues in PDHA1 and CPT2. Using a tryptic peptide library of purified PDHA1 and a Glu-C peptide library of purified CPT2, we found that PDHA1 lysine 336 (Supplementary information, Fig. S5a) and CPT2 lysine 457 and 458 (Supplementary information, Fig. S5b) were possibly lactylated (Lac-K336, Lac-K457/8). The presence of Lac-K336, as well as Lac-K457/8, was confirmed by matching the MS/MS spectra of synthetic Lac-K336- and Lac-K457/8-containing PDHA1 and CPT2 peptides to those from the peptide library (Supplementary information, Fig. S5a, b).

We assessed the ability of AARS2 to lactylate synthetic PDHA1 K336, CPT2 K457/8 peptides and intact PDHA1 and CPT2 proteins in vitro. As opposed to the AARS2^N104Y^ mutant, which has the asparagine 104 to tyrosine switch, equivalent to asparagine 71 to tyrosine switch in the AARS1 (AARS1^N71Y^, the inactivated form of AARS1^38^) (Supplementary information, Fig. S6a), AARS2 lactylated the synthetic K336-containing PDHA1 peptide as well as the K457/8-containing CPT2 peptide (Fig. 5b; Supplementary information, Fig. S6b) via lactate- and ATP-dependent and pyrophosphate-inhibitable mechanisms. However, AARS2 did not lactylate those peptides in which lysine residues had been changed to alanine (Supplementary information, Fig. S6c). These results suggested that the mechanism via which AARS2 attaches lactate to lysine is identical to that is used to attach alanine to lysine^27^ (Supplementary information, Fig. S6d). The *K*_*m*_ of AARS2 acting on a synthetic K336-containing PDHA1 peptide was within the physiological lactate levels of 5.21 mM, while the turnover number (*K*_*cat*_) of AARS2 for this peptide was 1.19 (Fig. 5c), indicating that AARS2 was an efficient lactyltransferase.

We generated polyclonal site-specific antibodies that recognized Lac-K336 (Supplementary information, Fig. S6e) and Lac-K457/8 (Supplementary information, Fig. S6f). Treatment with 10 mM Me-Lac increased the Lac-K336 and Lac-K457/8 levels of ectopically expressed PDHA1 (Fig. 5d) and CPT2 (Fig. 5e) in C2C12 and HL-1 cells (Supplementary information, Fig. S6g). However, the lactylation was not observed when *Aars2* was knocked out (Fig. 5d, e). Also, Lac-K336 and Lac-K457/8 increased in AARS2-overexpressing primary myoblasts and decreased after *Aars2* KO (Supplementary information, Fig. S6h). These results suggest that AARS2 acts as the lactyltransferase of PDHA1 K336 and CPT2 K457/8. Moreover, consistent with the observation that lactate can be transported into mitochondria by monocarboxylate transporter 1 (MCT1),^39^ Me-Lac supplementations in the culture media increased, whereas inhibition of MCT1 with α-cyano-4-hydroxycinnamic acid (α-CHCA) decreased mitochondrial lactate levels (Fig. 5f), and this result was consistent with α-CHCA treatment which reduced Lac-K336 and Lac-K457/458 levels in C2C12 cells (Fig. 5g, h). These results, together with the observation that mitochondrial lactate levels increased proportionally with lysate lactate levels, both are in the range of AARS2 *K*_*m*_ toward lactate, in mouse leg skeletal muscle after running (Fig. 5i), indicating that exercise-induced lactate may regulate mitochondrial lactylation.

Running enhanced Lac-K336 and Lac-K457/8 levels in mouse muscle cells (Fig. 5j), consistent with the finding that hypoxia increased both lysate and mitochondria lactate (Supplementary information, Fig. S6i) and Lac-K336 and Lac-K457/8 levels in C2C12 cells (Fig. 5k, l). PHD inhibition via roxadustat increased Lac-K336 and Lac-K457/8 (Supplementary information, Fig. S6j, k) in C2C12 cells, thereby highlighting the physiological significance of Lac-K336 and Lac-K457/8. Estimation via MS showed that Lac-K336 and Lac-K457/8 levels in mouse leg skeletal muscles were as high as 30% under simultaneous AARS2 overexpression and Me-Lac treatment (Fig. 5m), and reached 20% after mice running for 30 min (Fig. 5n) or when mice muscle exposed to hypoxia (Fig. 5o), indicating that a substantial portion of PDHA1 and CPT2 is lactylated under intracellular muscle cell hypoxia and high lactate in vivo, a condition that mimics endurance exercise.

Converting the K336 of PDHA1 and K457/8 of CPT2 to K336Q and 2 K/Q of the lactylation mimetic, glutamine (Q), reduced the activities of both enzymes while changing K336 and K457/8 to K336R and 2 K/R of the non-lactylation mimetic, arginine (R), negligibly affected their specific activities (Fig. 5p, q), indicating that lactylation inactivated PDHA1 and CPT2. Although AARS2 overexpression (Fig. 5p, q) and Me-Lac supplementation (Fig. 5r, s) increased Lac-K336 and Lac-K457/8 and inactivated PDHA1 and CPT2, they did not alter lactylation of lactylation site-null PDHA1 and CPT2 mutants, which was consistent with the observation that *Aars2*^*−/−*^ mouse leg muscle showed elevated PDC and CPT2 activities (Supplementary information, Fig. S6l), confirming that lactylation underlies AARS2-mediated PDHA1 and CPT2 inactivation.

Finally, physiologic level of alanine failed to inhibit K336 lactylation in vitro (Supplementary information, Fig. S6m); methyl-alanine supplementation negligibly affected Lac-K336 and Lac-K457/8 and the specific activities of PDHA1 and CPT2 (Supplementary information, Fig. S6n, o), eliminating the possibility that alanine affects lactylation, and protein alanylation, induced by AARS2 upregulation, regulates PDHA1 and CPT2 activities.

### SIRT3 reverses PDHA1 and CPT2 lactylation

**Fig. 6:**
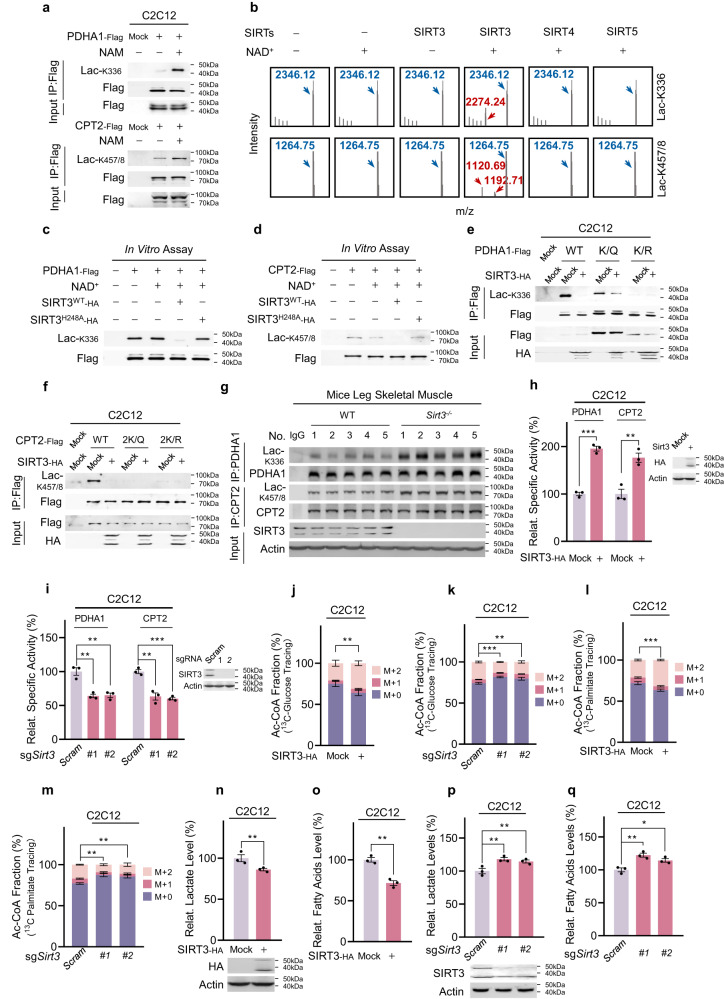
SIRT3 reverses Lac-K336 and Lac-K457/8. **a** NAM treatments increase Lac-K336 and Lac-K457/8. Lac-K336 and Lac-K457/8 levels of PDHA1 and CPT2 purified from C2C12 cells and NAM-treated C2C12 cells were detected. Cells were examined 3 h after 5 mM NAM treatment. **b** SIRT3 delactylates Lac-K336 and Lac-K457/8. The ability of SIRT3, SIRT4 catalytic domain and SIRT5 to delactylate synthetic Lac-K336- and Lac-K457/8-containing peptides was analyzed via MS to detect the formation of lactylated species. **c**–**f** SIRT3 decreases Lac-K336 and Lac-K457/8 levels. The Lac-K336 and Lac-K457/8 levels in purified PDHA1 (**c**) and CPT2 (**d**) were detected before and after being delactylated with SIRT3 or catalytic dead SIRT3^H248A^, and the SIRT3 expression effects on wild-type, lactylation-null PDHA1 (**e**), and CPT2 (**f**) were detected in C2C12 cells. **g** Ablation of *Sirt3* increases Lac-K336 and Lac-K457/8 levels in vivo. Lac-K336 and Lac-K457/8 levels of wild-type and *Sirt3*^*−/−*^ mouse leg skeletal muscles were detected (*n* = 5). **h**, **i** SIRT3 regulates PDHA1 and CPT2 activities. Relative activities of PDHA1 and CPT2 that were purified from C2C12 cells and SIRT3-overexpressing (**h**) or *Sirt3* KO (**i**) C2C12 cells were compared (*n* = 3). **j**–**m** SIRT3 regulates Ac-CoA production from glycolysis and FAO. The M + 0, M + 1, and M + 2 fractions of Ac-CoA from ^13^C-glucose (**j,** **k**) and ^13^C-palmitate (**l,** **m**) were detected in C2C12 cells either overexpressing SIRT3 (**j**, **l**) or in which *Sirt3* had been knocked out using independent sgRNAs (**k**, **m**) (*n* = 3). The chasing time for 10 mM ^13^C-glucose and 100 μM ^13^C-palmitate were 1 h and 12 h, respectively. **n**–**q** SIRT3 regulates lactate and free fatty acids levels. Lactate (**n**, **p**) and free fatty acid levels (**o,** **q**) in C2C12 cells overexpressing SIRT3 (**n**, **o**) or C2C12 cells in which *Sirt3* had been knocked out via independent sgRNAs (**p**, **q**) were detected (*n* = 3). All data are reported as mean ± SEM of three independent experiments. Statistical significance was assessed by unpaired two-tailed Student’s *t*-test and two-way ANOVA: *P < 0.05; **P < 0.01; ***P < 0.001.

To investigate whether Lac-K336 and Lac-K457/8 levels are dynamically regulated, we evaluated their responses to nicotinamide (NAM), a general inhibitor of sirtuins and deamidases that remove multiple amide-bonded protein modifications.^27^ NAM treatment increased Lac-K336 and Lac-K457/8 levels of ectopically expressed PDHA1 and CPT2 in C2C12 cells, respectively (Fig. 6a). Because Lac-K336 and Lac-K457/8 are mitochondrial protein modifications, we assessed the delactylase activities of mitochondrial sirtuins, namely SIRT3, SIRT4, and SIRT5. SIRT3 delactylated synthetic Lac-K336 and Lac-K457/8 peptides via an NAD^+^-dependent pathway, whereas the catalytic domain of SIRT4 or SIRT5 did not (Fig. 6b), indicating that SIRT3 is a delactylase for Lac-K336 and Lac-K457/8. This was further confirmed by the observation that both PDHA1 and CPT2 interacted with SIRT3 (Supplementary information, Fig. S7a, b), and that wild-type SIRT3 decreased Lac-K336 and Lac-K457/8 levels of purified intact PDHA1 (Fig. 6c) and CPT2 (Fig. 6d), whereas amidase-inactivated SIRT3^H248A^ mutant^27^ did not. Moreover, SIRT3 overexpression decreased Lac-K336 and Lac-K457/8 levels in wild-type C2C12 cells, but not in those carrying lactylation site-null PDHA1 and CPT2 mutants (Fig. 6e, f). Furthermore, PDHA1 and CPT2 purified from the leg skeletal muscles of *Sirt3* KO mice (*Sirt3*^*−/−*^ mice) showed higher Lac-K336 and Lac-K457/8 levels, respectively, than those from the leg muscles in wild-type mice (Fig. 6g).

The specific activities of PDHA1 and CPT2 expressed in SIRT3-overexpressing and *Sirt3* KO C2C12 cells were higher and lower, respectively, than those expressed in C2C12 cells (Fig. 6h, i). However, neither SIRT3 overexpression nor *Sirt3* KO affected the specific activities of lactylation site-null PDHA1 and CPT2 mutants (Supplementary information, Fig. S7c–f). Moreover, SIRT3 overexpression and *Sirt3* KO increased and decreased ^13^C-Ac-CoA production from ^13^C-glucose (Fig. 6j, k) and ^13^C-palmitate (Fig. 6l, m), respectively, in C2C12 cells.

Furthermore, SIRT3 overexpression decreased lactate (Fig. 6n) and free fatty acids levels (Fig. 6o) in C2C12 cells, whereas *Sirt3* KO increased these levels (Fig. 6p, q). These results were consistent with that SIRT3 delactylated and reversed the effects of lactylation on PDHA1 and CPT2. Lastly, SIRT3 overexpression, which may delactylate as well as deacetylate PDHA1,^18^ resulted in much more pronounced PDHA1 activation in C2C12 cells than in *Aars2* KO C2C12 cells, suggesting that lactylation is a major PDHA1 inactivating mechanism (Supplementary information, Fig. S7g).

### OXPHOS drives lactylation to feedback limit OXPHOS and endurance running ability

**Fig. 7:**
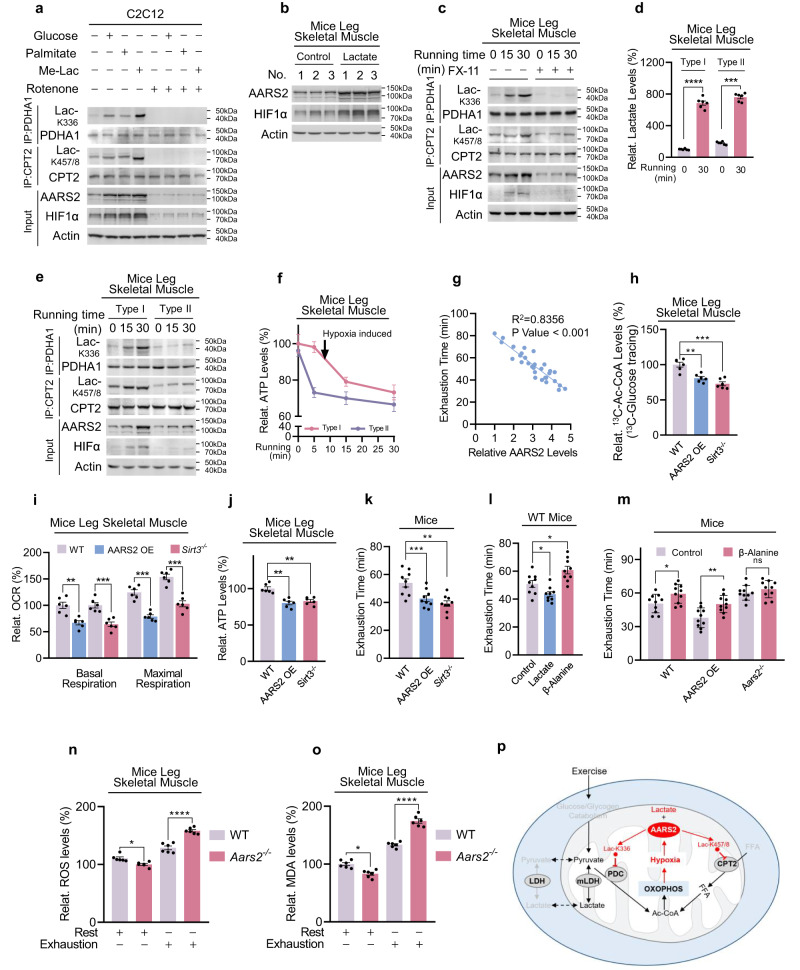
OXPHOS drives lactylation to feedback limit OXPHOS and endurance running ability. **a** OXPHOS induces intracellular hypoxia. HIF1α and AARS2 levels of C2C12 cells in DMEM base and glucose- (25 mM), palmitate- (200 μM) and Me-lac- (2 mM) supplemented DMEM media were tested in the presence or absence of 1 μM rotenone which inhibits OXPHOS. **b** Lactate injection induces mouse muscle hypoxia. HIF1α and AARS2 levels of mouse hind leg thighs were detected before and after 2 g/kg lactate was injected into gastrocnemius muscle (*n* = 3). **c** Lactate production is essential for inducing hypoxia and lactylation in mouse leg muscles. The HIF1α, AARS2, and lactylation levels of mouse hind leg thighs of untreated mice and mice pre-treated with FX-11 were assessed when they started to run. **d** Running leads to the accumulation of lactate in mouse leg skeletal muscles. Relative lactate levels in type I and type II mouse skeletal muscles were determined before and after 30 min of running (*n* = 6). **e** Running induces more pronounced lactylation in type I muscle. HIF1α and AARS2, as well as Lac-K336 and Lac-K457/8 levels in type I and type II mice skeletal muscles, were determined when started to run. **f** Drop of ATP in type I skeletal muscle is correlated with an increase in Lac-K336 and Lac-K457/8. ATP levels in type I and II mouse leg skeletal muscles were monitored after running for indicated times (*n* = 6). Changes in Lac-K336 and Lac-K457/8 are shown (Fig. 5j). **g** Basal AARS2 levels in mouse leg skeletal muscle are inversely correlated with their running exhaustion time. High-intensity running exhaustion time and resting leg muscle AARS2 levels were correlated (*n* = 30). **h** Lac-K336 and Lac-K457/8 inhibit the influx of mouse skeletal muscle Ac-CoA. The ^13^C-Ac-CoA levels in wild-type, AARS2 overexpressing and *Sirt3*^*−/−*^ mouse leg skeletal muscle were detected 1 h after each mouse received a ^13^C-glucose injection via the tail vein (*n* = 6). **i**, **j** Lactylation reduces mouse skeletal muscle OCRs and ATP production. OCR (**i**) and ATP levels (**j**) in mitochondria in the leg skeletal muscles of resting, AARS2 overexpressing, and *Sirt3*^*−/−*^ mice, were analyzed (*n* = 6). **k** Lac-K336 and Lac-K457/8 inhibit mouse running exhaustion time. Mouse high-intensity running exhaustion time was measured in wild-type, muscle AARS2-overexpressing, and *Sirt3*^*−/−*^ mice (*n* = 9). **l** Lactate and β-alanine inversely regulate mouse running exhaustion time. High-intensity running exhaustion times of untreated mice or treated with lactate or β-alanine via gastrocnemius muscle injection were measured, respectively (*n* = 9). **m** β-alanine prolongs mice exhaustion time in an AARS2-dependent manner. High-intensity running exhaustion times of wild-type, AARS2 overexpressing, and *Aars2*^*−/−*^ mice with or without gastrocnemius muscle β-alanine injection (*n* = 10) were measured. **n**, **o** Loss of lactylation results in higher ROS damage. ROS levels (**n**) and induced malondialdehyde levels (**o**) of wild-type and *Aars2*^*−/−*^ mice at the time of running exhaustion (*n* = 6) were compared. **p** Diagram showing that exercise promotes lactate oxidation through OXPHOS, which promotes intracellular hypoxia that induces AARS2 and Lac-K336 and Lac-K457/8 to feedback inhibit Ac-CoA influxes from lactate/glycolysis and FAO and OXPHOS. The mechanisms revealed in this study are marked by red arrows. All data are reported as mean ± SEM of three independent experiments. Statistical significance was assessed by unpaired two-tailed Student’s *t*-test and two-way ANOVA: *P < 0.05; **P < 0.01; ***P < 0.001; ****P < 0.0001; ns no significance.

In vitro hypoxia exposure of mouse leg tissues induced Lac-K336 and Lac-K457/8 in wild-type, but not in *Aars2*^*−/−*^ mouse leg tissues, indicating that hypoxia induces AARS2 as well as Lac-K336 and Lac-K457/8 (Supplementary information, Fig. S8a). To confirm that exercise-induced intramuscular hypoxia may be driven by activated muscle cell OXPHOS that acts as an oxygen sink,^1^ supplementation of culture media with either glucose, palmitate, or low lactate to enhance OXPHOS (Supplementary information, Fig. S8b) induced HIF1α alongside AARS2, Lac-K336 and Lac-K457/8 in C2C12 cells, whereas abrogating cell OXPHOS with rotenone diminished these effects (Fig. 7a). These results substantiated those of studies which demonstrated that enhancement of OXPHOS induced HIF1α in other cell types^40–43^ and confirmed that enhanced OXPHOS drives intramuscular hypoxia and lactylation. Moreover, re-oxygenating hypoxia-treated C2C12 cells not only decreased Lac-K336 and Lac-K457/8 levels, but also activated PDHA1 and CPT2 and increased Ac-CoA production (Supplementary information, Fig. S8c, d), consistent with that OXPHOS-induced intramuscular hypoxia is the driver of OXPHOS inhibition. However, re-oxygenation negligibly affected SIRT3 activity (Supplementary information, Fig. S8e), suggesting that OXPHOS promptly responds to AARS2-mediated lactylation but not SIRT3-mediated delactylation.

An injection of 2 g/kg lactate to mouse hind leg thigh muscle induced HIF1α, AARS2 (Fig. 7b), and Lac-K336/Lac-K457/8 (Supplementary information, Fig. S8f), while inhibition of LDHA using 2 mg/kg FX-11 decreased mouse muscle lactate production during running (Supplementary information, Fig. S8g) and largely abrogated running-linked induction of HIF1α, AARS2 and Lac-K336 and Lac-K457/8 in mouse hind leg thighs (Fig. 7c), echoing that LDHA inhibitor oxamate inhibits Lac-K336 and Lac-K457/8 in C2C12 cells (Supplementary information, Fig. S8h). These data highlight the fact that lactate production and oxidation play major roles in inducing muscle cell hypoxia and lactylation during exercise. Notably, 30 min running, which resulted in a substantial accumulation of lactate in both mitochondria-rich endurance ability-relative type I and mitochondria-less sprint ability-relative type II mouse skeletal muscles (Fig. 7d), induced more pronounced HIF1α, AARS2 and Lac-K336 and Lac-K457/8 in type I muscles (Fig. 7e), suggesting that lactylation regulates type I skeletal muscle OXPHOS and endurance exercise ability more than it regulates type II skeletal muscle OXPHOS and sprint ability. The observation that the ATP drop in type I muscle occurred coincidentally with Lac-K336 and Lac-K457/8 induction, as opposed to the ATP drop in type II muscle which occurred prior to Lac-K336 and Lac-K457/8 induction (Figs. 7f, 5j), supports this hypothesis. Moreover, wild-type, hypo-lactylated *Aars2*^*−/−*^, and hyper-lactylated *Sirt3*^*−/−*^mice all had comparable sprint abilities (Supplementary information, Fig. S8i), because lactate injection in mouse hind leg thigh only exerted a negligible effect on their sprinting abilities (Supplementary information, Fig. S8j). By contrast, mouse high-intensity running exhaustion times were moderately positively correlated with their basal leg muscle SIRT3 levels (Supplementary information, Fig. S8k) and strongly inversely related to their basal leg muscle AARS2 levels (Fig. 7g; Supplementary information, Fig. S8l). Moreover, AARS2 overexpressing specifically in mouse skeletal muscle by crossing h*AARS2* expression mice with *CKMM-cre* mice (Supplementary information, Fig. S8m) and knocking out *Sirt3*, both increased Lac-K336 and Lac-K457/8 (Fig. 6g; Supplementary information, Fig. S8n), but decreased Ac-CoA influx (Fig. 7h), OCR (Fig. 7i), and ATP levels (Fig. 7j) in leg skeletal muscles and shortened running exhaustion time (Fig. 7k). Furthermore, in contrast to lactate injection which shortened mouse running exhaustion time, β-alanine injection to hind leg thigh muscle decreased Lac-K336 and Lac-K457/8 (Supplementary information, Fig. S8f), consistent with that β-alanine was an alanine analog (Fig. 5a) that binds to the alanine-binding pocket of AARS2 (Supplementary information, Fig. S4f, g), thus decreasing Lac-K336 and Lac-K457/8 levels in vitro (Supplementary information, Fig. S9a, b), activating PDHA1 and CPT2 (Supplementary information, Fig. S9c, d), and increasing Ac-CoA levels (Supplementary information, Fig. S9e), OCR (Supplementary information, Fig. S9f), and ATP levels (Supplementary information, Fig. S9g) in C2C12 cells. β-alanine resulted in an increase in running exhaustion time in C57BL/6 mice (Fig. 7l) as well as in mice with intact AARS2, but not in mice lacking AARS2 (Fig. 7m). Lastly, the notion that Lac-K336 and Lac-K457/8 inhibit OXPHOS and constrain endurance running was further supported by the finding that both basal and maximum respiration of mouse leg skeletal muscles, were increased by *Aars2* KO, but decreased by *AARS2* overexpression during exercise, and the respiration responses were correlated to AARS2 levels (Supplementary information, Fig. S9h).

Possible physiologic roles played by Lac-K336 and Lac-K457/8 in exercise may be highlighted by examining Lac-K336- and Lac-K457/8-null *Aars2*^*−/−*^ mice. While *Aars2*^*−/−*^ mice endured extensive running and AARS2-overexpressing mice displayed impaired extensive running (Fig. 7m), *Aars2*^*−/−*^ mice accumulated higher levels of ROS and ROS-induced Malondialdehyde (MDA), lipid peroxidation and oxidative damage marker^44^ in their leg skeletal muscles than those in the leg skeletal muscles of wild-type mice, at the time of exhaustion (Fig. 7n, o). This suggested that muscle cells may use mitochondrial protein lactylation to protect against possible oxidative damage during endurance exercise. Thus, while enhanced OXPHOS is required to convert lactate-, glucose- and fatty acids-generated Ac-CoA to ATP during endurance exercise, Ac-CoA influx to OXPHOS is feedback inhibited by OXPHOS-induced intracellular hypoxia (Fig. 7p).

## Discussion

We demonstrated that OXPHOS is subject to negative regulation by mitochondrial protein lactylation, which is induced by OXPHOS-induced intracellular hypoxia. Therefore, mitochondrial protein lactylation represents a feedback mechanism to constrain OXPHOS. In addition to lactic acid binding to hydroxycarboxylic acid receptor 1^45^ and activating matrix metalloproteinase-2 to activate TGF-β signaling,^46^ AARS2-mediated lactylation represents another means of lactate signaling. Given that mitochondrial protein lactylation responds to intracellular hypoxia, its significances should at least include preventing drawbacks of hypoxia, such as ROS overproduction and ROS damage as observed in the current study. More physiologic or pathologic significances of mitochondrial protein lactylation are anticipated because high lactic acid and hypoxia responses occur in such physiologic processes as exercise and embryonic development, and in such pathologic processes as cancers.

Mitochondrial protein lactylation integrates metabolism and hypoxia, which are interconnected, to constrain OXPHOS. High lactic acid in embryonic and cancer development is due to an increased requirement for anabolites including lactic acid precursor pyruvate, and high lactic acid in exercise is due to enhanced glycolysis and glycogen catabolism-hiked pyruvate. Moreover, lactic acid can only be oxidized via OXPHOS, which consumes intracellular oxygen. These, together with that lactic acid levels vary substantially in these processes, make mitochondrial protein lactylation an efficient mechanism to constrain OXPHOS. Notably, although lactate can be a substrate of OXPHOS when intracellular oxygen is not depleted, mitochondrial protein lactylation takes effects to inhibit OXPHOS when intracellular hypoxia is induced, such as during the exhausting stage of endurance exercise. It is distinct from other OXPHOS-regulating mechanisms, such as acetylation and allosteric regulation of PDHA1, which integrate nutrients (acetyl-CoA) and hormonal signals^18,47^ to facilitate ATP supply after the start of exercise, during which intracellular hypoxia is not induced and the critical PO_2_ for maximal mitochondrial respiration is as low as 1–2 Torr^1,48^ to ensure a fully functional OXPHOS. We don’t exclude the possibility that mitochondrial protein lactylation may also regulate exercise ability by affecting other mitochondrion-rich and lactate-utilizing cells including heart cells, as we observed in cultured heart cells and given that AARS2 mutations have been linked to cardiomyopathy.^49^

AARS2 and SIRT3 are both known regulators of OXPHOS, lactate and muscle function. AARS2 mutation causes impaired OXPHOS, mitochondrial cardiomyopathy, muscle function loss and tissue lactate accumulation.^29–31,36^ SIRT3 loss causes impaired OXPHOS and lactate accumulation.^50,51^ Moreover, SIRT3 is induced during training,^50^ suggesting that SIRT3 may play a role in training adaption. SIRT3 has multiple roles in regulating OXPHOS. Under aerobic conditions, SIRT3 decreases PDHA1 acetylation to promote lactate production and FAO in mouse muscle,^51^ while under hypoxic conditions, it decreases PDHA1 lactylation to promote OXPHOS. Via either process, SIRT3 promotes muscle cell ATP production during exercise. Furthermore, decreasing mitochondrial protein lactylation by targeting both AARS2 and SIRT3 may temporarily boost muscle cell OXPHOS and exercise endurance as we observed in mice. However, these endurance exercise-boosting approaches should not be encouraged, as loss of mitochondrial protein lactylation protection may lead to ROS damage.

## Materials and methods

### Reagents and antibodies

Chemical and reagents: ATP (Cat# A6559), lactate (Cat# L6661), methy-lactate (Cat# 230340), β-alanine (Cat# 146064), L-alanine (Cat# A7627), DAPI (Cat# D9542), MG132 (Cat# M8699), PPi (Cat# 433314), NAM (Cat# 72340), ^13^C-palmitate (Cat# 605573) and cell counting kit-8 (Cat# 96992) were purchased from Sigma-Aldrich. Oligomycin A (Cat# S1478), FCCP (Cat# S8276), α-CHCA (Cat# S8612) and roxadustat (Cat# S1007) were purchased from Selleck. FX-11 (Cat# HY-16214) was purchased from MedChemExpress. Lambda protein phosphatase (Cat# P0753) was purchased from New England BioLabs. ^13^C-glucose (Cat# CLM1396) was purchased from Cambridge Isotope Labs. Protein-A sepharose beads (Cat# 16-156) was purchased from Merck Millipore. Penicillin-Streptomycin (Cat# 15070063) was purchased from Invitrogen. CellTiter-Glo® 2.0 reagent kit (Cat# G9241) was purchased from Promega. Lactate dehydrogenase activity colorimetric assay kit (Cat# K726) and free fatty acid quantification kit (Cat# K612) were purchased from Biovision. Pyruvate dehydrogenase (PDH) enzyme activity microplate assay kit (Cat# ab109902) and SIRT3 activity assay kit (Cat# ab156067) were purchased from Abcam. Synthetic peptides were purchased from Toppeptide. Synthetic oligonucleotides were purchased from Genepharma.

Antibodies: anti-AARS1 (Cat# sc165990) was purchased from Santa Cruz. Anti-VHL (Cat# ab140989), anti-HIF2α (Cat# ab109616), anti-HIF3α (Cat# ab2165), anti-CPT2 (Cat# ab110293), anti-PDHA1-pS293 (Cat# ab177461), anti-COXIV (Cat# ab202554), anti-GAPDH (Cat# ab1439) and anti-Actin (Cat# ab1440) were purchased from Abcam. Anti-AARS2 (Cat# 22696-1-AP), anti-PDHA1 (Cat# 18068-1-AP), anti-DLD (Cat# 16431-1-AP), anti-DLAT (Cat# 13426-1-AP), anti-PDHB (Cat# 14744-1-AP), anti-PHD1 (Cat# 12984-1-AP), anti-PHD2 (Cat# 19886-1-AP), anti-PHD3 (Cat# 18325-1-AP), anti-CPT1A (Cat# 15184-1-AP), anti-EHHADH (Cat# 26570-1-AP), anti-ACADS (Cat# 16623-1-AP), anti-ACADM (Cat# 55210-1-AP) and anti-ACADL (Cat# 17526-1-AP) were purchased from Proteintech. Anti-HIF1α (Cat# 36169S) was purchased from Cell Signaling Technology. Anti-PDHA1-pS232 (Cat# LS-C265964/64724) was purchased from LifeSpan Biosciences. Anti-PDHA1-pS300 (Cat# ABS194) was purchased from EMD Millipore Corporation. Anti-Laminin (Cat# L9393) was purchased from Sigma-Aldrich. Anti-Flag (Cat# M20008), anti-HA (Cat# M20002), anti-Myc (Cat# M20003) were purchased from Abmart. Goat anti-rabbit IgG (Cat# 111-035-003) and Goat anti-mouse IgG (Cat# 115-035-003) were purchased from Jackson. Anti-LacK336, anti-LacK457/8, anti-P377OH, and anti-ECHS1 were home-made in this study.

### Animals

Male C57BL/6 J mice were obtained from Shanghai SLAC Laboratory Animal Co., Ltd. (Shanghai, China). Mice were housed in cages under a 12 h light/dark cycle in a temperature- and humidity-controlled room, and fed standard laboratory chow and water ad libitum. They were then randomly divided into control and experimental groups. Conditional h*AARS2* transgenic mice were generated in a C57BL/6 genetic background by inserting CAG-LSL-h*AARS2*-WPRE-PA in the Rosa26 gene using a CRISPR-Cas9-mediated genome editing system by Shanghai Model Organisms Center, Inc. (Shanghai, China). Specific h*AARS2* transgenic mice were generated by crossing conditional h*AARS2* transgenic mice with *CKMM-cre* mice (Jackson Stock Number #029100). *Aars2*^flox/flox^ mice containing two loxP sites flanking exon1–5 were obtained from GemPharmatech Co., Ltd. (Shanghai, China). Muscle-specific transgenic mice were generated by crossing *Aars2* conditional KO mice with *CKMM-cre* mice. *Sirt3*^*−/−*^ mice were obtained from the Shanghai Model Organisms Center, Inc., and their genotypes were confirmed via PCR. All animal procedures were conducted in accordance with the animal care committee of Fudan University.

### Cell culture

Mouse myoblast C2C12, mouse cardiomyocytes HL-1 and human embryonic kidney HEK293T cell lines were purchased from the American Type Culture Collection (USA). All cell lines mentioned above were cultured in Dulbecco’s Modified Eagle’s Medium (DMEM) (Hyclone, USA) supplemented with 10% fetal bovine serum (Gibco, USA) at 37 °C under 5% CO_2_ in a humidified incubator. All cell lines were tested and authenticated by evaluating their morphology and growth rate; all were mycoplasma-free.

Mouse primary hepatocytes were isolated using a two-step perfusion method. Mice were intraperitoneally injected with 1% Nembutal, and their portal veins were catheterized. Subsequently, 0.5 mM EDTA and 50 IU/mL collagenase IV (Sigma Aldrich, USA) were preheated, and successively perfused into the liver until the liver structure disintegrated and cells dispersed. The cell suspensions, so obtained, were filtered through a 100-μm filter (NEST, Wuxi, China), washed with William’s E Medium (Gibco, USA), and purified using Percoll solution (GE, USA). Hepatocytes were seeded at a density of 10^6^ cells/well in serum-free William’s E Medium. Mouse primary hepatocytes were verified by evaluating their cell morphology and albumin expression. Mouse primary myoblasts were isolated and the culturing condition was modified from published protocols.^52^ The hind limb muscles were isolated from adult mice and then digested in DMEM containing 400 U/mL collagenase II (Sigma Aldrich, USA) and 2.5% HEPES. The suspensions were filtered through 70-μm and then 30-μm filters. Cells were suspended in F-10 medium containing 20% FBS and 10 ng/mL basic fibroblast growth factor (bFGF) in the 10% Matrigel pre-coated dish, kept at 37 °C for 72 h. Cells were then transfered to non-Matrigel coated dish for 45 min to remove fibroblasts which adhered in this step. The cell supernatant was transfered to a new Matrigel pre-coated dish and cultured.

### Site-specific antibodies

Lactylation site-specific polyclonal antibodies were generated in 3-month-old male New Zealand rabbits using synthetic Lac-K336-containing PDHA1 peptide or Lac-K457/8-containing CPT2 peptide. These peptides were conjugated through a C-terminal cysteine to keyhole limpet hemocyanin (KLH). Rabbits were immunized with 250 μg of the conjugate in Freunds complete adjuvant. Booster injections were performed every 4 weeks. The rabbits were exsanguinated 10–14 days after the final boost. 50 mL blood collected from each rabbit was centrifuged. The supernatant (serum) was purified with antigen peptides according to published protocols. Hydroxylation site-specific polyclonal antibodies were generated with OH-P377-containing AARS2 peptide as described above. Antigen used:

Lac-K336-containing PDHA1 peptide: ASVEEL(K-Lac)EIDVEV-C

Lac-K457/8-containing CPT2 peptide: EFLK(K-Lac)QKLS-C

OH-P377-containing AARS2 peptide: C-GSLV(P-OH)VVVET

### Plasmid constructs

*AARS2* and *VHL* were cloned into pcDNA3.1(b+)-Flag and pcDNA3.1(b+)-Myc, respectively, between *Xho*I and *EcoR*I sites. *PDHA1* and *CPT2* were cloned into pcDNA3.1(b+)-Flag between *Xho*I and *EcoR*I sites. *PDH2* was cloned into pcDNA3.1(b+)-Myc between *Xho*I and *EcoR*I sites, and *SIRT3* was cloned into pcDNA3.1(b+)-HA between *Xho*I and *EcoR*I sites.

### Cell transfection

Plasmid transfections were performed using polyethylenimine (PEI, Polyscience, USA) or Lipofectamine 8000 (Beyotime, Shanghai, China). For PEI transfection, the plasmid and PEI were added into serum-free DMEM with vigorous shaking, and the mixture was incubated for 15 min before being added into the cell culture medium. For Lipofectamine 8000 transfection, the plasmid and Lipofectamine 8000 were added into serum-free DMEM, mixed gently with a pipette, and then immediately added to the cell culture medium. The culture medium was replaced every 12–16 h after transfection. Cells were harvested after 36 h.

### Cell treatments

#### Me-lactate, alanine, β-alanine, and NAM

Cells were cultured in a standard RMPI 1640 medium (US Biological, USA), and transferred to amino acid-free RMPI 1640 for 30 min before treatments. Indicated concentrations of Me-lactate, alanine, and β-alanine were added into the culture media when cell density reached 50%–60% confluence at 2–4 h before harvest. NAM treatment consisted of adding 5 mM NAM to serum-free DMEM medium 3–6 h before harvest.

#### Rotenone

Rotenone was added to culture media to obtain a 1 μM solution 20 min before cell harvesting.

#### α-CHCA

Monocarboxylate transporter inhibitor α-CHCA (5 mM, final concentration) was added to the culture medium 2 h before harvesting.

#### Roxadustat

Roxadustat (50 μM, final concentration) was added to the culture medium 6 h before harvesting.

#### Hypoxia

Cells were cultured under normoxia before being transferred to a hypoxia incubator (ThermoFisher Scientific, USA) with a gas mixture containing 5% CO_2_ and 1% O_2_ balanced with nitrogen. Cells were harvested at the indicated time points that were not longer than 8 h after hypoxic exposure. Chamber oxygen levels were monitored at sampling times.

### Isolation of cell mitochondria

A hypotonic buffer containing 20 mM HEPES (pH 7.5), 140 mM KCl, 10 mM EDTA, and 5 mM MgCl_2_ with a protease and phosphatase inhibitor cocktail was added to cells after washing with PBS. The cells were then scraped and broken with a syringe. The homogenate was centrifuged at 800× *g* for 10 min to separate the nucleus and other debris. The supernatant was then centrifuged at 12,000 × g and 4 °C for 30 min. The obtained supernatant consisted of plasma. Following this, the precipitate was washed thrice with a hypertonic buffer containing 20 mM HEPES (pH 7.5), 800 mM KCl, 10 mM EDTA, 5 mM MgCl_2_, in PBS. The final precipitate contained mitochondria. The purity of plasma and mitochondria was verified using plasma and mitochondria markers, Actin, and COXIV, via western blot assays.

### Mouse injections

#### Gastrocnemius muscle injection

Lactate, alanine, and β-alanine were prepared in 0.9% saline to obtain a final concentration of 500 mM, the pH of which was adjusted to 7.4. A 2.0 g/kg dose of each drug was intramuscularly injected to the mice gastrocnemius muscle. A similar volume of 0.9% saline was injected into the same muscles as a control.

#### Intravenous MG132 injection

MG132 was prepared in 0.9% saline to obtain a final concentration of 500 mM, the pH of which was adjusted to 7.4. Mice were injected intravenously with 1 mg/kg MG132 twice a week for 4 weeks before being used for experimentation and muscles were sampled for detection.

#### FX-11 injection

Mice were intraperitoneally injected with 2% DMSO as a control or 2 mg/kg DMSO-dissolved LDHA inhibitor FX-11 daily for 2 weeks, following which the mouse running tests were carried out.

### Mouse sprint and high-intensity exercise fatigue test

Mouse sprint performance test was modified from published protocols^52^: First, 20-week-old mice were placed on a ZH-PT/5 S treadmill equipped with a rear electrical stimulus grid set that delivered a 0.2 mA electric shock. Mice were first warmed up on the treadmill for 3 min at 6 m/min. Speed was increased to 18 m/min for 30 s. Afterward, we increased the speed from 18 m/min to 32 m/min with an increment of 2 m/min, with each increment lasting 15 s (total 15 × 8 = 120 s). The pattern was repeated with increasing speed until the mice could no longer keep up with the speed and gave up at the grid.

Mouse high-intensity exercise fatigue test was modified from published protocols.^53,54^ Briefly, 20-week-old mice were acclimated to the treadmill 2 days prior to the experiments by running at 5 m/min for 5 min and 10 m/min for 5 min followed by 15 m/min for 1 min every day. For the exercise experiments, speed was increased 5 m/min every 5 min until 20 m/min was reached, at which point the incline was increased 5 inclines every 5 min until exhaustion.

### Mouse leg fast and slow muscle preparation

Muscle preparation was modified from published protocols.^55^ Briefly, mice were euthanized and the soleus (SOL) muscles, mainly composed of slow muscle (type I), and the extensor digitorum longus (EDL), mainly composed of fast muscle (type II), were dissected and subjected to the following tests.

### Muscle hypoxia perfusion

Mice were euthanized and their leg skeletal muscles were dissected and immediately placed in Krebs Ringer solution (120 mM NaCl, 23.8 mM NaHCO_3_, 10 mM D-glucose, 4.8 mM KCl, 2.5 mM CaCl_2_, 1.2 mM KH_2_PO_4_, 1.2 mM MgSO_4_, 5 mM HEPES) in a water-jacketed bath of the 1200 A Isolated Intact Muscle Test System (Aurora Scientific, Canada), and subsequently aerated with or without 95% O_2_ and 5% CO_2_ at 25 °C for 30 min.

### Muscle tissue mitochondria isolation

Mouse leg muscles were dissected and rinsed in ice-cold mitochondrial isolation buffer containing 70 mM sucrose, 210 mM mannitol, 5 mM MOPS, 5 mM KH_2_PO_4_, 1 mM EGTA, and 0.5% (w/v) fatty acid-free bovine serum albumin (pH 7.2). The tissues were then homogenized with a tissue grinder (Shanghai Jing Xin, Shanghai, China) at 4 °C. The homogenate was centrifuged at 800× g and 4 °C for 10 min to remove nuclei and cell debris. The supernatant enriched in mitochondria was then centrifuged at 8000× *g* and 4 °C for 10 min. The mitochondria precipitate so obtained was used for assays.

### PO_2_ assay

Arterial blood was immediately collected in anticoagulation tubes from aorta abdominalis of mice that had run for indicated times. PO_2_ was determined using IDEXX VetStat (IDEXX B.V., The Netherlands) Electrolyte and a Blood Gas Analyzer.

### ROS assay

Mouse leg skeletal muscle ROS assays were conducted using a ROS Assay Kit (Cat# E-BC-K138-F, Elabscience Biotechnology Inc., Houston, TX, USA) according to the manufacturer’s instructions. The tissues were digested into a single-cell suspension via collagenase digestion. Then, 10^6^ cells were incubated with 10 μM DCFH-DA reagent for 30 min. Following centrifugation at 1000 rpm for 5 min, the cell pellets were washed twice and resuspended in Reagent 3 before being analyzed in a FACS Calibur flow cytometer.

### MDA assays

Lipid peroxidation was assessed using an MDA assay Kit (ab233471, Discovery Drive, Cambridge Biomedical Campus, Cambridge, CB2 0AX, UK) according to the manufacturer’s instructions. For tissue extract preparation, 50 mg fresh skeletal muscle was rapidly homogenized on ice in 500 μL 20 mM Na Phosphate buffer (pH 3–3.2) with 0.5% TritonX-100 followed by centrifugation at 10,000× *g* for 15 min at 4 °C. Insoluble material was removed, and the supernatant was stored on ice for later assays. Prior to being used, all materials and prepared reagents were equilibrated to room temperature with gentle agitation. Next, 50 μL test samples, a blank control (dilution buffer), and serially diluted MDA standards were added to a 96-well clear bottom microplate. This was followed by incubating the reaction mixture at room temperature for 10–30 min. Then, 40 μL of Reaction Solution was added to bring the total assay volume up to 100 μL/well. The final reaction mixture was incubated at room temperature for 30–60 min. End-point absorbance was measured using an absorbance plate reader with path-check correction at OD_695_.

### Muscle tissue immunofluorescence

Paraffin sections for the leg skeletal muscles of wild-type, muscle-specific AARS2 overexpressing mice, and mice that had run were prepared. Sections were deparaffinized, rehydrated, antigen retrieved in EDTA antigen retrieval buffer (pH 8.0), and blocked with 3% BSA for 30 min. Nuclei were stained with DAPI. HIF1α, pS293-PDHA1, AARS2, and COXIV were stained with their primary and secondary antibodies, respectively. Images of the prepared sections were captured under a fluorescence microscope.

### Muscle tissue λ-phosphatase treatment

The mice were sacrificed and 50 mg fresh skeletal muscle was rapidly homogenized on ice in 400 μL lysis buffer containing 0.5% NP-40, 50 mM Tris-HCl (pH 7.5), and 150 mM NaCl, and centrifuged at 10,000× *g* for 15 min at 4 °C, following which insoluble material was removed. The supernatant was then collected on ice for the subsequent assay. Next, 400 units of λ-phosphatase (New England Biolab) and 10 mM MnCl_2_ were added to the supernatant and incubated at 30 °C for 30 min, and the supernatant was used for PDHA1 activity assay.

### Small RNA interference

Oligonucleotides used for small interfering RNA (siRNA)-mediated silencing of *VHL*, *PHD1*, *PHD2*, *PHD3*, *AARS2*, and *SIRT3* were synthesized by GenePharma (Shanghai, China). Oligonucleotides were transfected into cells 24–36 h before being harvested. Knockdown efficiency was verified using western blott assays. The siRNA sequences used for targeting genes are as follows:

*Phd1* siRNA: 5′-ACUCAUUGGUUCCUUUAAGGG-3′

*Phd2* siRNA: 5′-UGGAUUUGUACCAUUCUUCUG-3′

*Phd3* siRNA: 5′-UCGAAACUCUGAAGAGAAGGG-3′

### Gene KO by CRISPR/Cas9

To knock out *Phd2*, *Vhl*, *Aars2*, *Cpt2*, and *Sirt3* in C2C12 cells, CRISPR/Cas9 protocols were employed. Guide RNA containing the target sequence was transfected into C2C12 cells, which were then selected after 36 h using 2 µg/mL puromycin (Amresco, USA) for 3 days. The cells that eventually survived were harvested and seeded into a 96-well plate at a density of 1 cell/well. Successful KO of each gene was verified via western blot assays. The guide sequences are as follows:

sg*Phd2* #1:

F: 5′-CACCGACACCGGCAAGTTCACGGAC-3′

R: 5′-AAACGTCCGTGAACTTGCCGGTGTC-3′

sg*Phd2* #2:

F: 5′-CACCGCAAGTTCACGGACGGGCAGC-3′

R: 5′-AAACGCTGCCCGTCCGTGAACTTGC-3′

sg*Aars2* #1:

F: 5′-CACCGCTGCTAAGTACCGGGGCCGT-3′

R: 5′-AAACACGGCCCCGGTACTTAGCAGC-3′

sg*Aars2 #2*:

F: 5′-CACCGCGAGCGGCTGCGCTTTGACG-3′

R: 5′-AAACCGTCAAAGCGCAGCCGCTCGC-3′

sg*Cpt2* #1:

F: 5′-CACCGATCGTCCCGGGCGGCAATGG-3′

R: 5′-AAACCCATTGCCGCCCGGGACGATC-3′

sg*Cpt2* #2:

F: 5′-CACCGAGGCGCGGCATCATCGTCCC-3′

R: 5′-AAACGGGACGATGATGCCGCGCCTC-3′

sg*Vhl* #1:

F: 5′-CACCGCGTTCCAATAATGCCCCGGA-3′

R: 5′-AAACTCCGGGGCATTATTGGAACGC-3′

sg*Vhl* #2:

F: 5′-CACCGCCGATCTTACCACCGGGCAC-3′

R: 5′-AAACGTGCCCGGTGGTAAGATCGGC-3′

sg*Sirt3* #1:

F: 5′-CACCGCCAGTACAGACAGGGCAGCGGGG-3′

R: 5′-AAACCCCCGCTGCCCTGTCTGTACTGGC-3′

sg*Sirt3* #2:

F: 5′-CACCGCTTTCAACAAACCTCCAGGGAGG-3′

R: 5′-AAACCCTCCCTGGAGGTTTGTTGAAAGC-3′

### Immunoprecipitation

Cells were lysed using lysis buffer containing 0.5% NP-40 buffer (50 mM Tris-HCl, pH 7.5), 150 mM NaCl, 0.5% Nonidet P-40, 1 μg/mL aprotinin, 1 μg/mL leupeptin, 1 μg/mL pepstatin, and 1 mM (PMSF). Tissues were homogenized in the lysis buffer before lysis. Affinity antibody beads were added to the lysate to enrich target proteins. The enriched proteins on beads were washed with 0.5% NP-40 buffer thrice before being mixed with SDS-PAGE loading buffer to release proteins for analysis.

### Western blot assay

Cultured cells were lysed and boiled with SDS-PAGE loading buffer. Mouse tissues were homogenized using lysis buffer containing 0.5% NP-40 buffer, 50 mM Tris-HCl (pH 7.5), 150 mM NaCl, 0.5% Nonidet P-40, and a mixture of protease inhibitors (Sigma-Aldrich), before being boiled with 5× loading buffer. After centrifugation at 12,000 rpm and 4 °C for 15 min, the supernatant of the lysates was collected for western blot assay according to standard procedures. Western blot signals were detected using the Typhoon FLA 9500 (GE Healthcare, Little Chalfont, UK).

### Dot blot assay

Peptides were diluted with ddH_2_O at the indicated dilutions. Samples were blotted onto nitrocellulose membranes, and allowed to air dry at room temperature. The membrane was blocked with 5% milk, and then incubated with a primary antibody for peptide recognition and a secondary antibody for detection. Blotting signals were detected using a Typhoon FLA 9500 (GE, USA).

### Ubiquitination assay

For ubiquitination analysis, HA-tagged ubiquitin and target plasmids were co-transfected into cells. After 36 h of transfection, MG132 was added to the medium for 4–6 h before harvesting. Cells were collected, lysed, and boiled in 1% SDS buffer (Tris-HCl, pH 7.5, 0.5 mM EDTA, 1 mM DTT) for 10 min. Immunoprecipitation was performed in 10-fold diluted lysates with 0.5% NP-40 buffer, and ubiquitination was analyzed following standard western blot protocols.

### PDHA1 activity assay

PDHA1-Flag and PDHB-Myc were co-expressed in indicated cells. Cells were lysed on ice using 0.5% NP-40 buffer supplemented with protease inhibitors. PDHA1 was immuno-purified using flag beads (Sigma Aldrich) and then washed thrice with 0.1% NP-40 buffer. PDHB co-purified with PDHA1 was quantified via Myc-western blot and used to normalize the activity of PDHA1. The PDHA1 activity assay was performed as previously described.^56^ Briefly, a reaction buffer containing 50 mM KH_2_PO_4_ (pH 7.0), 1 mM MgCl_2_, 2 mM sodium pyruvate, 0.2 mM thiamin diphosphate, and 0.1 mM 2,6-dichlorophenolindophenol (2,6-DCPIP) was added to the purified PDHA1/PDHB complex to initiate the reaction. The reactions were maintained at 30 °C. The reaction was monitored by measuring the reduction of 2,6-DCPIP at 600 nm on a Roche spectrophotometer (Basel, Switzerland).

### PDC activity assay

The enzyme activity of the PDC complex was measured using a PDH Enzyme Activity Microplate Assay Kit (Abcam, USA) according to the manufacturer’s instructions. Cells and mouse tissues were harvested to extract proteins. Samples were loaded on the plate and incubated at RT for 3 h. The assay solution was added to each well, and PDH activity was determined by following the reduction of NAD^+^ to NADH, coupled with the reduction of a reporter dye to yield a colored (yellow) reaction product, the concentration of which was monitored by measuring absorbance at 450 nm.

### CPT2 activity assay

CPT2 activity was assayed by measuring the decrease in CoA levels. The reactions were conducted in a 300 μL reaction mix containing 50 mM Tris-HCl (pH 7.5), 120 mM KCl, 1 mM EDTA, 2 mM CoA, and 2 mM Palmity-L-Carnitine. Subsequently, CPT2 was immunoprecipitated from HEK293T cells. The assay was initiated in a thermostatic oscillation incubator at 1000 rpm and 37 °C, and the mixture was collected at 0 min, 5 min, 10 min, 20 min, and 30 min. The reaction was terminated by adding 50 μL of 10 mM DTNB. The supernatant was monitored using a microplate reader at 410 nm.

### LDH activity assay

An LDH Activity Colorimetric Assay Kit (Biovision, USA) was used. Briefly, 1 × 10^6^ cells were homogenized with ice-cold assay buffer, incubated on ice for 10 min, and centrifuged at 10,000× *g* for 15 min. Next, the supernatant was transferred to a fresh tube, and 100 μL of the reaction mixture (10 μL sample, 40 μL assay buffer, and 50 μL reaction mix) was transferred to a 96-well plate. Absorbance was immediately measured at 450 nm in kinetic mode at 37 °C for 0–20 min. LDH activity was measured based on the change in absorption during a specific time frame.

### CPT1A activity assay

CPT1A activity in cell homogenates was assayed spectrophotometrically by following the release of CoA-SH from palmitoyl-CoA using the general thiol reagent DTNB. Reaction mixtures containing DTNB buffer and cell homogenate were incubated at room temperature for 20 min to eliminate all the reactive thiol groups. After incubation, the absorbance was measured at 412 nm. To start the reaction, palmitoyl-CoA (100 µM, final concentration) and L-carnitine solution (1 mM, final concentration in 1 M Tris, pH 8.0) were added to the reaction mixtures. Reaction mixtures were incubated for 10 min at 37 °C. After incubation, the absorbance was measured at 412 nm. The difference between absorbance with and without substrates measures the release of CoA-SH. Activity was defined as nmole CoA-SH released/min/mg protein. The protein content of the cell homogenates was determined according to the method of Bradford.

### SIRT3 activity assay

SIRT3 Activity Assay Kit (Abcam, USA) was used according to the manufacturer’s instructions. The reaction mixture contains assay buffer, Fluoro-Substrate peptide, NAD, developer, and samples. Read fluorescence intensity for 30 min to 60 min at 1 min to 2 min intervals using microtiter plate fluorometer with excitation at 340–360 nm and emission at 440–460 nm. Measure and calculate the rate of reaction while the reaction velocity remains constant.

### Blood lactate

The blood lactate concentration in tail vein blood was measured using a Lactate Scout4 (SensLab GmbH, Germany). The tail of each mouse was cut using a blade, and the first droplet of blood was wiped away. The tail was gently pressed, and the second droplet of blood was sufficient to fill the measurement chamber of the lactate sensor enabling the concentration to be determined.

### Cell and tissue OCR assays

OCR assays were performed using the OROBOROS Oxygraph-2K module (Oroboros Instruments GmbH, Austria). Oxygen consumption values were normalized to the cell number or mitochondrial content.

For cultured cells, approximately 1 × 10^6^ cells were digested and resuspended in PBS. The basal respiratory rate was determined with the substrate (5 mM pyruvate and 0.1 mM malate). The ATP production capacity and maximal respiration were successively determined using Oligomycin A and FCCP (Selleck, China) treatments, respectively.

For mouse muscle samples, mitochondria were isolated according to a published protocol and the isolated mitochondria samples were resuspended in respiration buffer (0.5 mM EDTA, 3 mM MgCl_2_, 60 mM lactobionic acid, 20 mM taurine, 10 mM KH_2_PO_4_, 20 mM HEPES, 110 mM D-sucrose and 0.1% w/v fatty acid-free BSA, pH 7.2).

### AARS2 kinetic assay

The pGEX-6p-1-AARS2 vector was transformed into the *Escherichia coli* strain, BL21 (DE3). The transformed cells were then induced with 0.2 mM IPTG when OD_600_ was 0.6–0.8. After cultured overnight at 16 °C, the bacteria were collected, homogenized in a buffer containing 50 mM Tris-HCl and 150 mM NaCl, and centrifuged at 12,000× *g* and 4 °C for 30 min. The supernatant was loaded onto an AKTA Purifier (GE, USA) with a His-Ni column. The concentration of protein so obtained was measured using the BSA protein assay kit.

The kinetic parameters of AARS2 were determined by measuring the lactylation of peptide substrates (PDHA1: MVNSNLASVEELKEIDVEVR; CPT2: EFLKKQKLS). *K*_*cat*_s and *K*_*m*_s of lactate were estimated by varying lactate levels from 1–10 mM, while peptide substrates were fixed at 0.5 mg/mL. Lactylation reactions were carried out in a 30 μL reaction mix containing 50 mM pH 7.5 HEPES, 25 mM KCl, 2 mM MgCl_2_, 1–10 mM lactate, 4 mM ATP, and 10 nM AARS2. Lactylated peptide produced was determined using MS with known concentrations of synthetic peptides (PDHA1: MVNSNLASVEELK_*Lac*_EIDVEVR; CPT2: EFLK_*Lac*_K_*Lac*_QKLS) as internal controls. *K*_*cat*_s and *K*_*m*_s were deduced using the Michaelis-Menten equation.

### ITC

Protein-small molecule interaction was determined using MicroCal PEAQ-ITC (Malvern Panalytical, UK) at 25 °C. Lactate, alanine, and β-alanine were dissolved in 50 mM Tris-HCl and 150 mM NaCl at a concentration of 300 μM. Recombinant AARS2 was diluted to 50 μM, and then added to the sample cell. Molecules were dropped into the sample cell every 2 min for 20 times.

### In vitro lactylation reactions

In vitro lactylation reactions were performed in a 30 μL reaction mix containing 50 mM HEPES (pH 7.5), 25 mM KCl, 2 mM MgCl_2_, 10 mM lactate, 4 mM ATP, 10 nM AARS2, and 0.05 mg/mL synthetic substrate peptide. The final pH of the reaction mixture was adjusted to 7.5 before AARS2 was added. The reaction was allowed to continue for 3 h at 37 °C. The peptide was desalted using C18 ZipTip (Millipore, USA), and subjected to analysis using a MALDI-TOF/TOF mass spectrometer (SCIEX-5800, AB Sciex, Framingham, MA, USA). Synthetic peptides sequences are as follows:

Synthetic PDHA1 peptide: Ac-MVNSNLASVEELKEIDVEVR

Synthetic PDHA1 K336A peptide: Ac-MVNSNLASVEELAEIDVEVR

Synthetic CPT2 peptide: Ac-EFLKKQKLS

Synthetic CPT2 K457/8 A peptide: Ac-EFLAAQALS

### In vitro de-lactylation reaction

In vitro de-lactylation reactions were conducted in a 30 μL reaction mix containing 50 mM HEPES (pH 7.5), 6 mM MgCl_2_, 1 mM DTT, 1 mM NAD^+^, 0.05 mg/mL synthetic lactylated peptide or recombinant PDHA1 and CPT2 protein, 1 mg/mL SIRT3, and 1 mM PMSF. The reaction was allowed to continue for 4 h at 37 °C. The peptide was desalted by passing it through a C18 ZipTip before subjecting it to analysis using a MALDI-TOF/TOF mass spectrometer (SCIEX-5800). The resulting PDHA1 and CPT2 were subjected to western blot analysis and enzyme activity assay. Synthetic peptides sequences are as follows:

Synthetic PDHA1 K336 peptide: MVNSNLASVEELK_*Lac*_EIDVEVR

Synthetic CPT2 K457/8 peptide: EFLK_*Lac*_K_*Lac*_QKLS

### MS/MS identification of AARS2 P377OH, PDHA1Lac-K336, and CPT2 Lac-K457/8

AARS2 or CPT2 proteins that were overexpressed in cells were affinity purified and digested with endoproteinase Glu-C. Overexpressed PDHA1 from C2C12 cells were affinity-purified and digested with trypsin. Hydroxylated or lactylated peptides were analyzed via MS/MS sequencing.

LC peaks eluted at the same time as those of standard synthetic peptides were subjected to MS analysis with an Orbitrap Fusion mass spectrometer (ThermoFisher Scientific, USA). Peptide counting and/or sample areas were compared to those of standard synthetic peptides. The MS machine was set in a data-dependent mode to switch automatically between MS and MS/MS acquisition. Survey full-scan MS spectra (m/z 350–1600) were acquired in the Orbitrap with a mass resolution of 60,000 at m/z 200. The AGC target was set to 300,000, and the maximum injection time was 50 ms. MS/MS acquisition was performed in Orbitrap with a cycle duration of 3 s, at a resolution of 15,000 at m/z 200. The intensity threshold was 50,000, and the maximum injection time was 200 ms. The AGC target was set to 200,000, and the isolation window was 2 m/z. Ions with charge states of 2+, 3+, and 4+ were sequentially fragmented via a high-energy collisional dissociation with a normalized collision energy of 30%, and the fixed first mass was set as 120. In all cases, one micro scan lasting 30 s was recorded using dynamic exclusion. Quantification of the targeted lactylated peptide was achieved via MS quantitation. Briefly, a ratio of lactylated peptide signal (the total ion counts (TIC) of lactylated form) to the total peptide signal (TIC of lactylated form + TIC of non-lactylated form) was calculated according to the following equation: TICK-Lac/(TICK-Lac + TICnon-K-Lac) = Ratio of K-Lac (RK-Lac). Synthetic peptides sequences are as follows:

Synthetic AARS2 peptide: ILKAPPGFLGSLVPVVVE

Synthetic AARS2 P377OH peptide: ILKAPPGFLGSLVP_*OH*_VVVE

Synthetic PDHA1 peptide: MVNSNLASVEELKEIDVEVR

Synthetic PDHA1 K336 peptide: MVNSNLASVEELK_*Lac*_EIDVER

Synthetic CPT2 peptide: FLKKQKLSPD

Synthetic CPT2 K457/8 peptide: FLK_*Lac*_K_*Lac*_QKLSPD

### LC-MS/MS quantitation of AARS2 P377OH, PDHA1Lac-K336, and CPT2 lac-K457/8

AARS2, PDHA1, and CPT2 were ectopically expressed in cultured C2C12 cells overexpressing AARS2 or treated with lactate. Cells were harvested and lysed in 0.1% NP-40 buffer (50 mM Tris-HCl, pH 7.5, 150 mM NaCl, 0.1% Nonidet P-40), and anti-FLAG M2 magnetic beads (Sigma-Aldrich) were used to precipitate PHD2, PDHA1, and CPT2 for 3 h at 4 °C. The beads were then washed twice with 0.1% NP-40 buffer, twice with ddH_2_O, and thrice with 50 mM NH_4_HCO_3_, which was followed by on-bead trypsin digestion overnight at 37 °C. The resulting peptides in supernatants were collected via centrifugation and dried in a speed vacuum (Eppendorf, Hamburg, Germany). The obtained peptides were stored at –80 °C for LC-MS/MS analysis. A previously published method^57^ was used to quantify targeted lactylated peptides. Briefly, a ratio of lactylated peptide signal (the total ion counts (TIC) of lactylated form) to the total peptide signal (TIC of lactylated form + TIC of non-lactylated form) was calculated according to the following equation: TICK-Lac/(TICK-Lac + TICnon-K-Lac) = Ratio of K-Lac (RK-Lac).

### Metabolite assay with LC-MS/MS

Approximately 1 × 10^7^ cells were treated with cold aqueous methanol solution (80% v/v) to quickly stop cell metabolism. Cells were scraped off from tubes, and placed at −80 °C overnight. For mouse muscle samples, approximately 80 mg of mouse leg muscle was homogenized in a cold aqueous methanol solution (80% v/v) at 4 °C. Samples were centrifuged at 15,000× *g* and 4 °C for 15 min, and supernatants were collected. The supernatants were then lyophilized and re-dissolved in 500 μL methanol/water (10:90 v/v). The separated metabolites were obtained using high-performance liquid chromatography with an LC-20AB pump (Shimadzu, Kyoto, Japan) and the Luna NH_2_ column (P/N 00B-4378-B0; 5 μm, 50 × 2.0 mm; Phenomenex, Torrance, CA, USA). The mobile phase comprised eluent A (0.77 g NH_4_OAc, 1.25 mL NH_4_OH, 25 mL ACN, and 300 µL acetic acid dissolved in 500 mL water) and eluent B. The elution program was as follows: 0.1 min, 85% B; 3 min, 30% B; 12 min, 2% B; 15 min, 2% B; and 16–28 min, 85% B. The flow rate of the pump was 0.3 mL/min, and the mass spectrometer used was the 4000 QTRAP system (AB Sciex, Framingham, MA, USA) operated in multiple reaction monitoring modes. The metabolite ions were monitored at: PYR 87-43; LAC 89-43; Ac-CoA 810-60; ATP 509-79; and AKG 145-101. Each measurement was obtained at least in triplicate.

### Metabolic flux assay

Metabolic flux experiments were performed when cells reached 90% confluence. The medium for ^13^C glucose labeling experiments contained 10 mM (U-^13^C) glucose (Cambridge Isotope Labs, MA, USA) and 2 mM unlabeled glutamine (Sigma-Aldrich, USA). Medium for ^13^C glutamine labeling experiments contained 10 mM unlabeled glucose and 2 mM (U-^13^C) glutamine (Cambridge Isotope Labs). Medium for ^13^C palmitate labelling experiments contained 0.1 mM (U-^13^C) palmitate (Sigma-Aldrich). After treatment, cells were washed thrice with PBS and subsequently treated with pre-cold methanol (80% v/v). At the same time, parallel dishes were used to count cell numbers. Metabolite extractions were then analyzed using LC-MS with a C18 column. Relative metabolite abundances were determined by normalizing the abundances of each metabolite to the internal standard and cell number. The incorporation of ^13^C atoms was denoted as m + n, where n was the number of ^13^C atoms.

### Structural modeling

Since no substrate-binding structures in human AARS2 have been observed to date, we modeled the structure of the alanine binding region with Phyre2 (Structural Bioinformatics Group, Imperial College, London). Prediction of the structure indicated that it was a high structural homolog of AARS2 in *Aquifex aeolicus*, which is an alanyl-tRNA synthetase (alias: alaS, PDB code: 1YFS) complexed with alanine. Thus, we further docked β-alanine and lactate into the complex structure and superimposed the modeled AARS2 to that of alaS from *Aquifex aeolicus*.

### Statistical methods

Statistical analysis was performed using Prism 8.0 (GraphPad Software, Inc., San Diego, CA, USA) and Excel (Microsoft Corp., Redmond, CA, USA). Pooled results were expressed as the mean ± SEM. Comparisons between groups were made via unpaired two-tailed Student’s *t*-test and two-way ANOVA. Statistical significance was set at *P* ≤ 0.05; ns no significance; *P < 0.05; **P < 0.01; ***P < 0.001; ****P < 0.0001.

### Supplementary information

Supplementary information, Fig. S1

Supplementary information, Fig. S2

Supplementary information, Fig. S3

Supplementary information, Fig. S4

Supplementary information, Fig. S5

Supplementary information, Fig. S6

Supplementary information, Fig. S7

Supplementary information, Fig. S8

Supplementary information, Fig. S9

Supplementary information, Table. S1
