# Supplementary material for: Hypoxia induces mitochondrial protein lactylation to limit oxidative phosphorylation
Source: Cell Res. 2024 Jan 2;34(1):13–30. doi: 10.1038/s41422-023-00864-6 (PMC10770133; doi:10.1038/s41422-023-00864-6)
Supplement: Supplementary file 1 — Supplementary information, Fig. S1 [file 41422_2023_864_MOESM1_ESM.pdf]

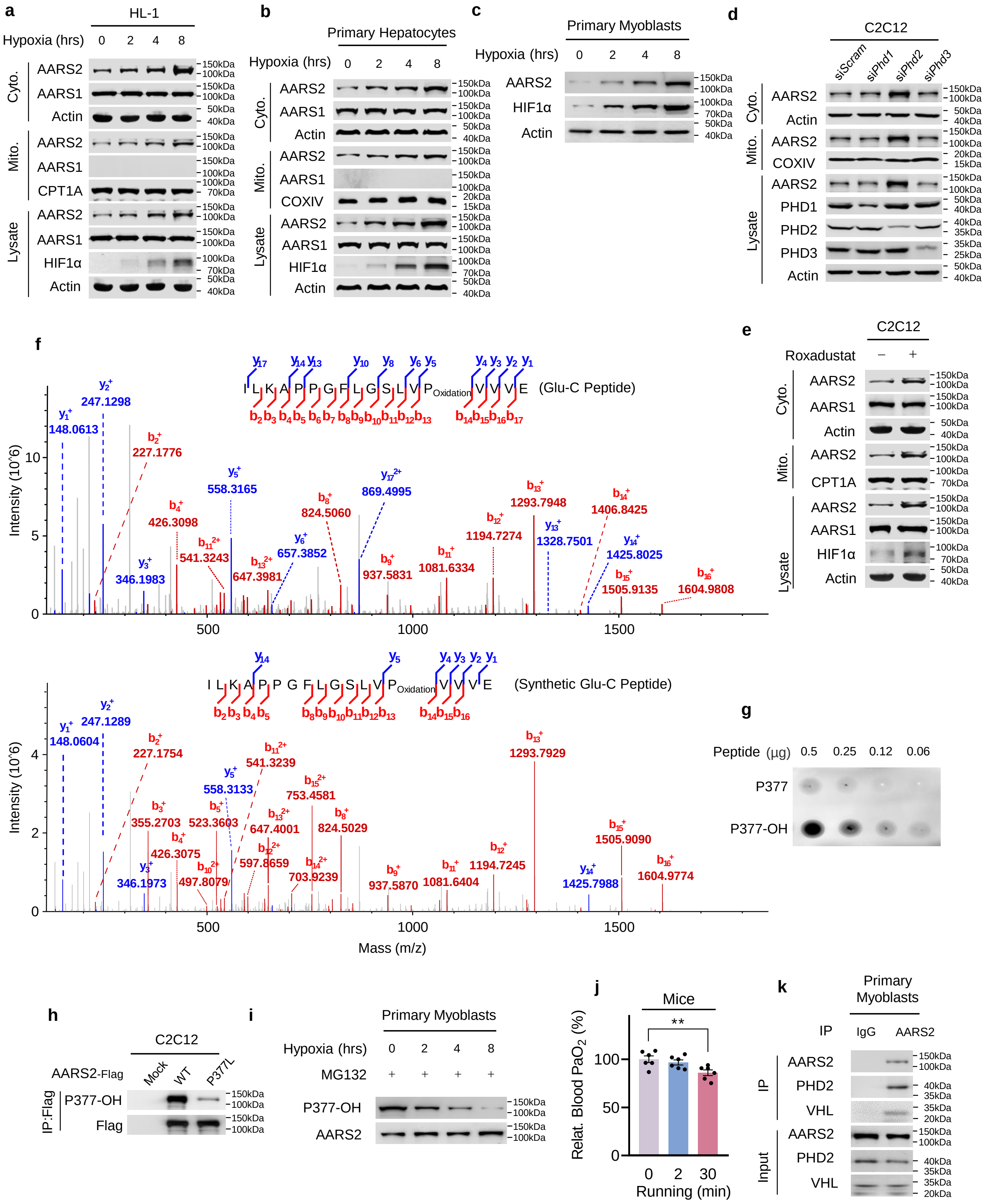

a
HL-1
Hypoxia (hrs) 0 2 4 8
Cyto. AARS2 AARS1 Actin
Mito. AARS2 AARS1 CPT1A
Lysate AARS2 AARS1 HIF1α Actin
150kDa 100kDa 50kDa 40kDa 70kDa
b
Primary Hepatocytes
Hypoxia (hrs) 0 2 4 8
Cyto. AARS2 AARS1 Actin
Mito. AARS2 AARS1 COXIV
Lysate AARS2 AARS1 HIF1α Actin
150kDa 100kDa 50kDa 40kDa 20kDa 15kDa 70kDa
c
Primary Myoblasts
Hypoxia (hrs) 0 2 4 8
AARS2 HIF1α Actin
150kDa 100kDa 70kDa 50kDa 40kDa
d
C2C12
siScram siPhd1 siPhd2 siPhd3
Cyto. AARS2 Actin
Mito. AARS2 COXIV
Lysate AARS2 PHD1 PHD2 PHD3 Actin
150kDa 100kDa 50kDa 40kDa 20kDa 15kDa 35kDa 25kDa
e
C2C12
Roxadustat – +
Cyto. AARS2 AARS1 Actin
Mito. AARS2 CPT1A
Lysate AARS2 AARS1 HIF1α Actin
150kDa 100kDa 50kDa 40kDa 70kDa
f
I L K A P P G F L G S L V P Oxidation V V V E (Glu-C Peptide)
Intensity (10^6)
y1+ 148.0613
y2+ 247.1298
b2+ 227.1776
b4+ 426.3098
y3+ 346.1983
b11 2+ 541.3243
y5+ 558.3165
b13 2+ 647.3981
y6+ 657.3852
b8+ 824.5060
y17 2+ 869.4995
b9+ 937.5831
b11+ 1081.6334
b12+ 1194.7274
b13+ 1293.7948
y13+ 1328.7501
b14+ 1406.8425
y14+ 1425.8025
b15+ 1505.9135
b16+ 1604.9808
I L K A P P G F L G S L V P Oxidation V V V E (Synthetic Glu-C Peptide)
y1+ 148.0604
y2+ 247.1289
b2+ 227.1754
b3+ 355.2703
b4+ 426.3075
y3+ 346.1973
b10 2+ 497.8079
b5+ 523.3603
y5+ 558.3133
b11 2+ 541.3239
b12 2+ 597.8659
b13 2+ 647.4001
b14 2+ 703.9239
b15 2+ 753.4581
b8+ 824.5029
b9+ 937.5870
b11+ 1081.6404
b12+ 1194.7245
b13+ 1293.7929
y14+ 1425.7988
b15+ 1505.9090
b16+ 1604.9774
500 1000 1500
Mass (m/z)
g
Peptide (µg) 0.5 0.25 0.12 0.06
P377
P377-OH
h
C2C12
AARS2-Flag Mock WT P377L
IP:Flag P377-OH Flag
150kDa 100kDa
i
Primary Myoblasts
Hypoxia (hrs) 0 2 4 8
MG132 + + + +
P377-OH AARS2
150kDa 100kDa
j
Mice
Relat. Blood PaO2 (%)
**
0 2 30
Running (min)
k
Primary Myoblasts
IP IgG AARS2
IP AARS2 PHD2 VHL
Input AARS2 PHD2 VHL
150kDa 100kDa 40kDa 35kDa 20kDa

Supplementary information, Fig. S1 PHD2-VHL proteasomal machinery regulates AARS2

a, b AARS2 protein levels in mouse HL-1 cardiomyocytes, primary hepatocytes are regulated by hypoxia. The total, mitochondrial, and cytosolic AARS2 levels in mouse HL-1 cardiomyocytes (**a**) and primary hepatocytes (**b**), were measured after hypoxia was induced in a hypoxia chamber for indicated durations.

c AARS2 protein levels in mouse primary myoblasts are regulated by hypoxia. AARS2 and HIF1α levels were detected in mouse primary myoblasts cultured in a hypoxia chamber for indicated time durations.

d, e PHD inhibition elevates AARS2 protein levels. The endogenous total, mitochondrial, and cytosolic AARS2 levels in C2C12 cells and *Phd1*, *Phd2*, and *Phd3* silenced C2C12 cells (**d**), as well as C2C12 cells that were untreated or treated with 50 μM of the PHD inhibitor, roxadustat for 6 h (**e**) were measured.

f MS-based identification of P377 as a hydroxylation site. MS/MS spectra that matched the projected P377OH-containing AARS2 Glu-C peptide (upper) and MS/MS spectra generated from the synthetic P377OH-containing AARS2 Glu-C peptide (lower) are shown.

g, h Verification of P377OH antibody. Reactivity of the P377-OH antibody towards P377- and P377OH-containing AARS2 tryptic peptides (**g**), as well as AARS2 and $AARS2^{P377L}$ (**h**), were determined by employing dot blot and western blot, respectively.

i Hypoxia regulates P377 hydroxylation in mouse primary myoblasts. P377OH levels in mouse primary myoblasts cultured in a hypoxia chamber for indicated time durations in the presence of MG132 to prevent proteasomal degradation were measured.

j Running-induced blood hypoxia in mice. Relative blood partial pressure of oxygen (PaO_2) levels in the blood of mice were detected before and after 2 and 30 min of running (n=6).

k AARS2 interacts with VHL and PHD2 in mouse primary myoblasts. The interaction between endogenous AARS2 and VHL and PHD2 in mouse primary myoblasts was detected via co-immunoprecipitation.

All data are reported as mean ± SEM of three independent experiments. Statistical significance was assessed by unpaired two-tailed Student's t-test: $^{**}P < 0.01$.
