# Supplementary material for: Hypoxia induces mitochondrial protein lactylation to limit oxidative phosphorylation
Source: Cell Res. 2024 Jan 2;34(1):13–30. doi: 10.1038/s41422-023-00864-6 (PMC10770133; doi:10.1038/s41422-023-00864-6)
Supplement: Supplementary file 2 — Supplementary information, Fig. S2 [file 41422_2023_864_MOESM2_ESM.pdf]

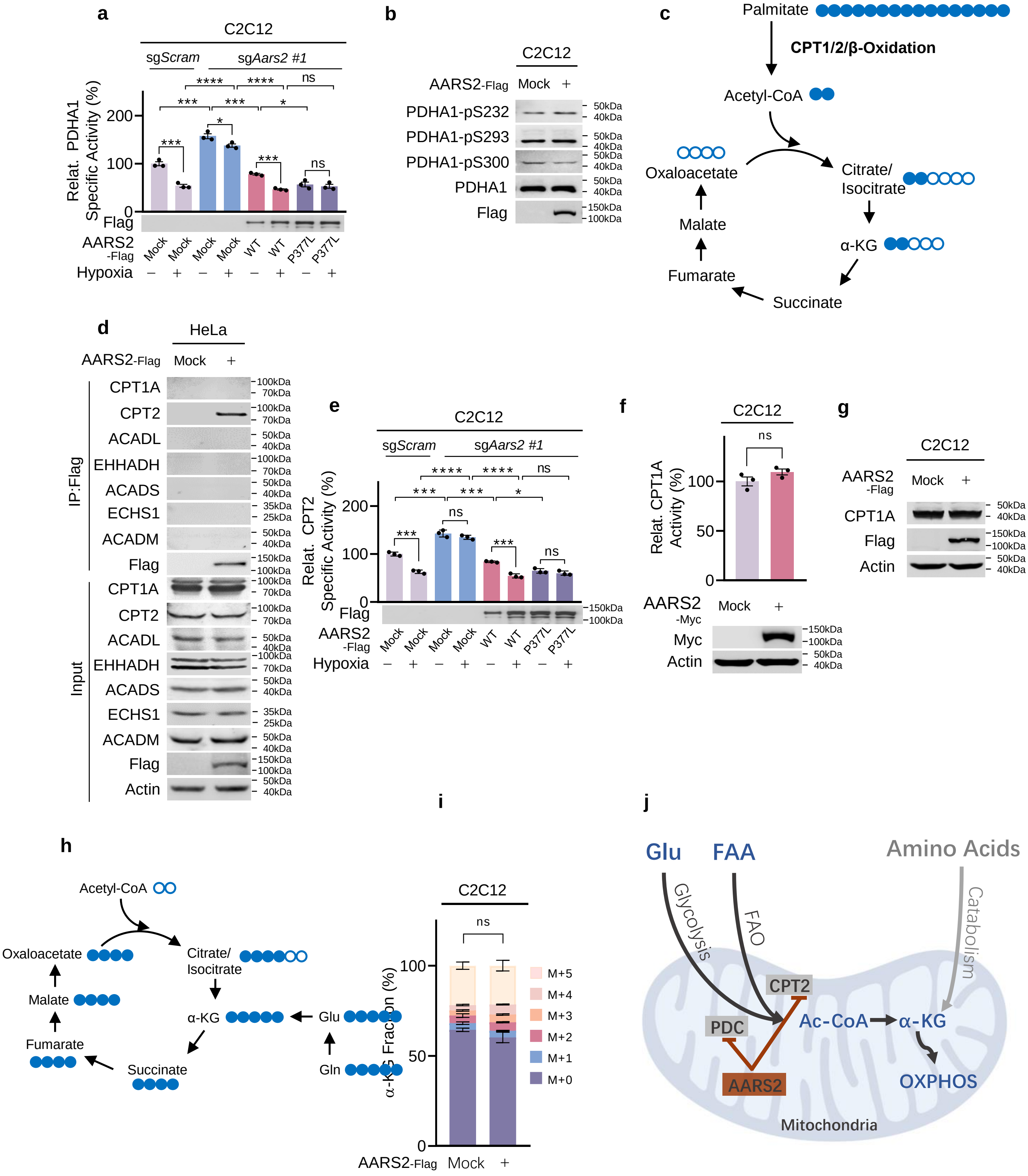
a
C2C12
sgScram
sgAars2 #1
Relat. PDHA1 Specific Activity (%)
Flag
AARS2 -Flag
Mock Mock Mock Mock WT WT P377L P377L
Hypoxia
b
C2C12
AARS2-Flag Mock +
PDHA1-pS232
PDHA1-pS293
PDHA1-pS300
PDHA1
Flag
c
Palmitate
CPT1/2/β-Oxidation
Acetyl-CoA
Oxaloacetate
Citrate/ Isocitrate
Malate
α-KG
Fumarate
Succinate
d
HeLa
AARS2-Flag Mock +
IP:Flag
Input
CPT1A
CPT2
ACADL
EHHADH
ACADS
ECHS1
ACADM
Flag
Actin
e
C2C12
sgScram
sgAars2 #1
Relat. CPT2 Specific Activity (%)
Flag
AARS2 -Flag
Hypoxia
f
C2C12
Relat. CPT1A Activity (%)
AARS2 -Myc Mock +
Myc
Actin
g
C2C12
AARS2 -Flag Mock +
CPT1A
Flag
Actin
h
Acetyl-CoA
Oxaloacetate
Citrate/ Isocitrate
Malate
α-KG
Glu
Gln
Fumarate
Succinate
i
C2C12
α-KG Fraction (%)
M+5
M+4
M+3
M+2
M+1
M+0
AARS2-Flag Mock +
j
Glu
FAA
Amino Acids
Glycolysis
FAO
Catabolism
CPT2
PDC
Ac-CoA
α-KG
AARS2
OXPHOS
Mitochondria

**Supplementary information, Fig. S2 AARS2 inhibits the influx of Ac-CoA from glycolysis and FAO**

**a** AARS2 hydroxylation mediates hypoxia-induced PDHA1 inactivation. The effects of *Aars*2 KO and either wildtype or P377L mutant reintroducing into *Aars*2 KO C2C12 cells on PDHA1 activities and their responses to hypoxia had been detected.

**b** AARS2 expression does not alter PDHA1 phosphorylation. Phosphorylation of PDHA1 serine 232, 293, and 300 in C2C12 cells and C2C12 cells overexpressing AARS2 were determined.

**c** Schematic of the conversion of $^{13}$C-labeled palmitate to $^{13}$C-Ac-CoA and TCA cycle intermediates.

**d** CPT2 interacts with AARS2. The interaction between ectopically expressed AARS2 and endogenous CPT1A, CPT2, and FAO enzymes was detected via co-immunoprecipitation.

**e** AARS2 hydroxylation mediates hypoxia-induced CPT2 inactivation. The effects of *Aars*2 KO and either wildtype or P377L reintroducing into *Aars2* KO C2C12 cells on CPT2 activities and their responses to hypoxia had been detected..

**f, g** AARS2 overexpression does not alter CPT1A activity. The specific activities (**f**, n=3) and protein levels (**g**) of CPT1A in C2C12 and AARS2-overexpressing C2C12 cells were compared.

**h** Schematic of the conversion of $^{13}$C-labelled glutamine to $^{13}$C-labeled $\alpha$-KG and TCA cycle intermediates.

**i** AARS2 does not affect $\alpha$-KG production from glutamine. Unlabeled and differently $^{13}$C-labeled labeled (M+0-M+5) $\alpha$-KG levels were detected in C2C12 cells and AARS2 overexpressing C2C12 cells after being labeled with 2 mM $^{13}$C-glutamine for 1 h (n=3).

**j** Schematic showing that AARS2 inhibits the glycolytic and FAO Ac-CoA influx but not $\alpha$-KG influx from amino acid catabolism for the TCA cycle.

All data are reported as mean $\pm$ SEM of three independent experiments. Statistical significance was assessed by unpaired two-tailed Student's t-test and two-way ANOVA: *$P < 0.05$; ***$P < 0.001$; ****$P < 0.0001$; ns no significance.
