# Supplementary material for: Hypoxia induces mitochondrial protein lactylation to limit oxidative phosphorylation
Source: Cell Res. 2024 Jan 2;34(1):13–30. doi: 10.1038/s41422-023-00864-6 (PMC10770133; doi:10.1038/s41422-023-00864-6)
Supplement: Supplementary file 3 — Supplementary information, Fig. S3 [file 41422_2023_864_MOESM3_ESM.pdf]

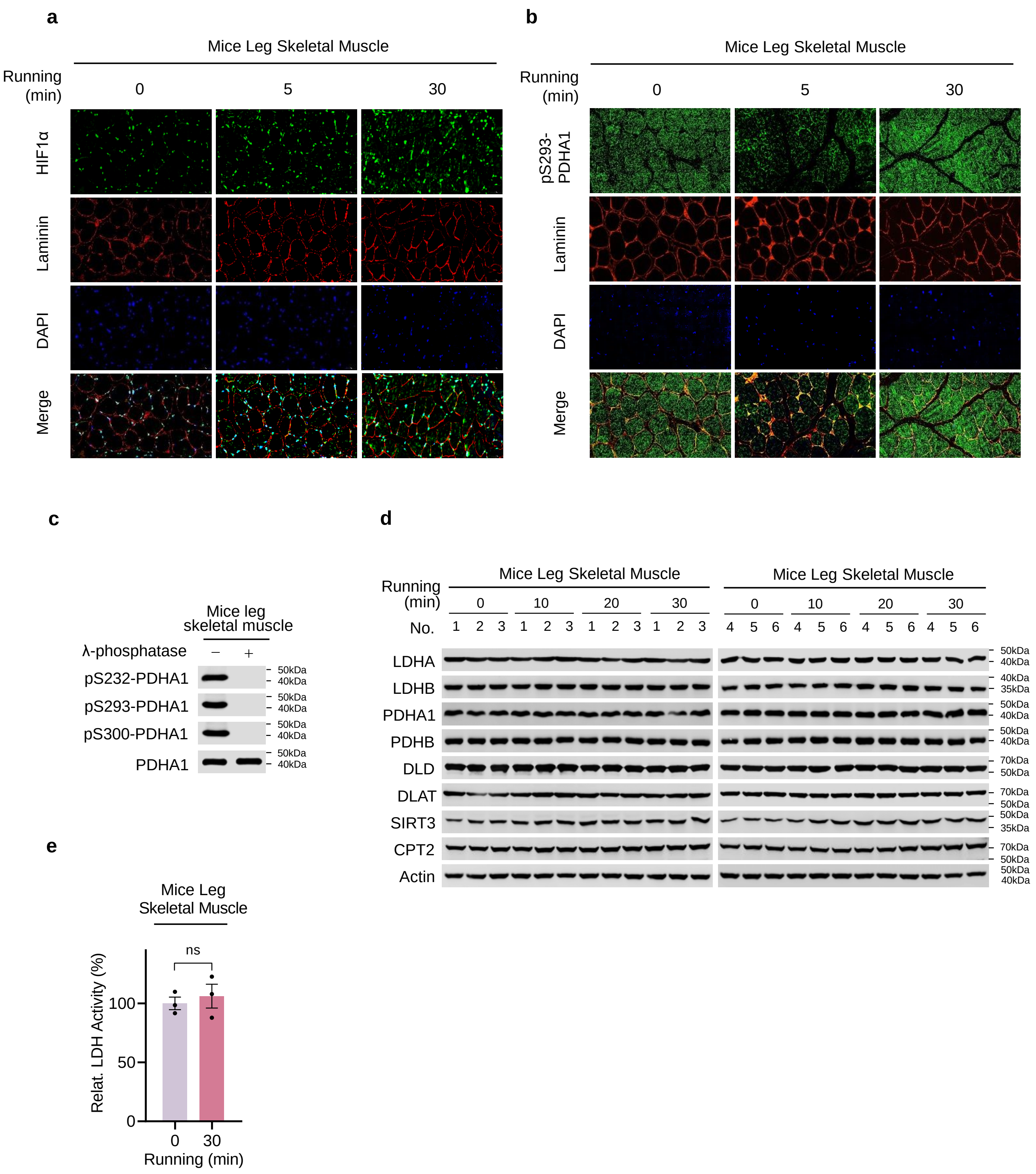
a
Mice Leg Skeletal Muscle
Running (min)
0
5
30
HIF1α
Laminin
DAPI
Merge
b
Mice Leg Skeletal Muscle
Running (min)
0
5
30
pS293-PDHA1
Laminin
DAPI
Merge
c
Mice leg skeletal muscle
λ-phosphatase
−
+
pS232-PDHA1
pS293-PDHA1
pS300-PDHA1
PDHA1
50kDa
40kDa
d
Mice Leg Skeletal Muscle
Mice Leg Skeletal Muscle
Running (min)
0
10
20
30
No.
1 2 3 1 2 3 1 2 3 1 2 3
4 5 6 4 5 6 4 5 6 4 5 6
LDHA
LDHB
PDHA1
PDHB
DLD
DLAT
SIRT3
CPT2
Actin
50kDa
40kDa
35kDa
70kDa
e
Mice Leg Skeletal Muscle
ns
Relat. LDH Activity (%)
100
50
0
0
30
Running (min)

Supplementary information, Fig. S3 Running induces AARS2 and lactate and inactivates PDHA1 and CPT2

a Running induces hypoxia in mouse leg skeletal muscle. Immunofluorescence staining of HIF1α in leg skeletal muscle sections of resting mice and mice that had run for 5 or 30 min, laminin was included as control.

b Running alters PDHA1 phosphorylation in mouse leg skeletal muscle. Immunofluorescence staining of pS293-PDHA1 in dissected leg skeletal muscle sections of resting mice and mice that had run for 5 or 30 min , laminin was included as control.

c λ phosphatase treatment diminishes the phosphorylation of PDHA1 of mouse leg muscle lysate. Phosphorylation of PDHA1 serine 232, 293, and 300 in mouse leg muscle lysates before and after λ phosphatase treatment was determined.

d Running does not alter lactate metabolism-related protein levels. The protein levels of LDHA, LDHB, PDHA1, PDHB, DLD, DLAT, SIRT3, and CPT2 (n=6) in the leg muscles of mice allowed to run for indicated durations were detected.

e Running does not alter LDH activity. Total LDH activities in the leg muscles of mice were determined before and after running for 30 min (n=3).

All data are reported as mean $\pm$ SEM of three independent experiments. Statistical significance was assessed by unpaired two-tailed Student’s t-test: ns no significance.
