# Supplementary material for: Hypoxia induces mitochondrial protein lactylation to limit oxidative phosphorylation
Source: Cell Res. 2024 Jan 2;34(1):13–30. doi: 10.1038/s41422-023-00864-6 (PMC10770133; doi:10.1038/s41422-023-00864-6)
Supplement: Supplementary file 4 — Supplementary information, Fig. S4 [file 41422_2023_864_MOESM4_ESM.pdf]

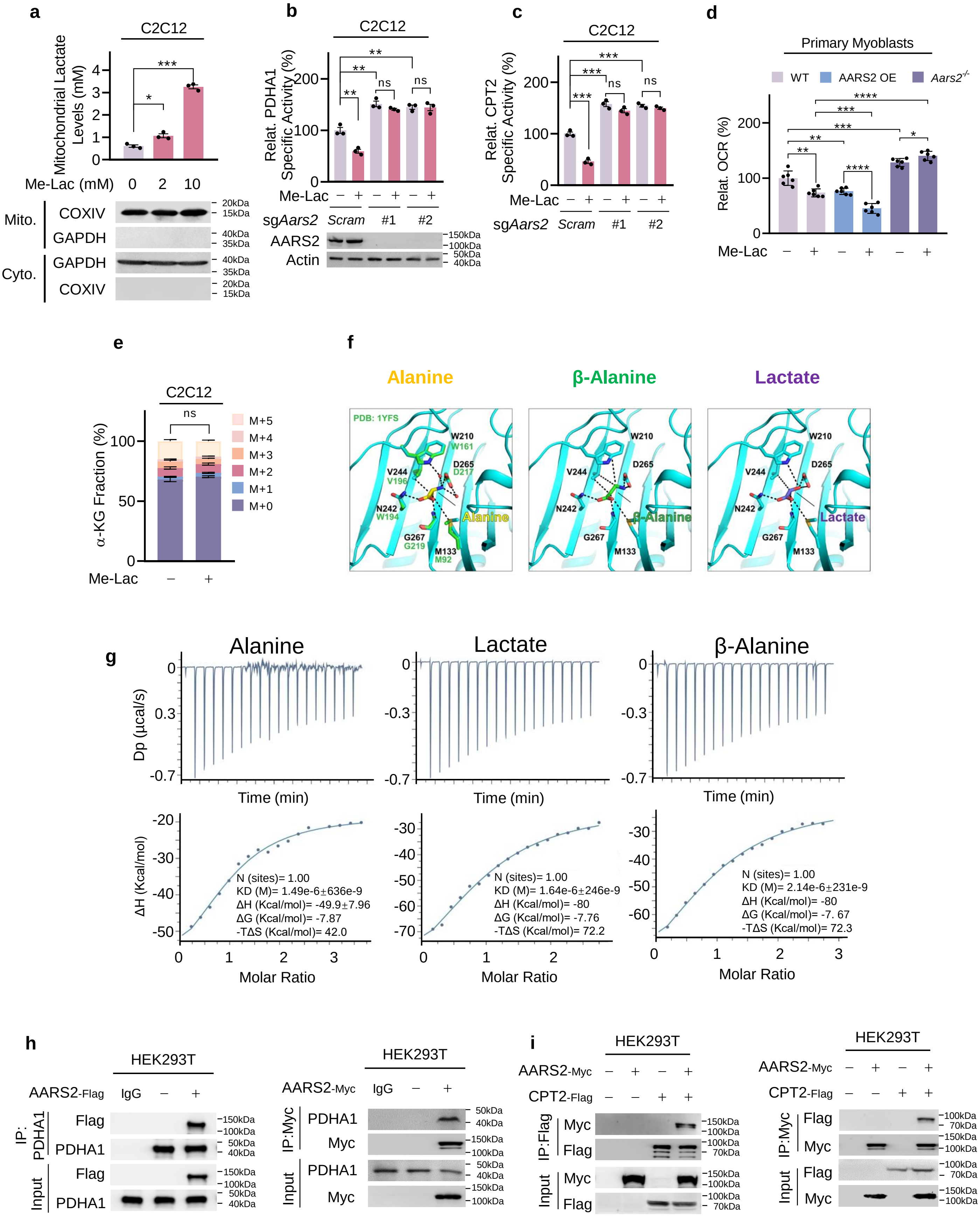

a
C2C12
Mitochondrial Lactate Levels (mM)
Me-Lac (mM) 0 2 10
Mito.
COXIV
GAPDH
Cyto.
GAPDH
COXIV
20kDa
15kDa
40kDa
35kDa
b
C2C12
Relat. PDHA1 Specific Activity (%)
Me-Lac
sgAars2 Scram #1 #2
AARS2
Actin
150kDa
100kDa
50kDa
40kDa
c
C2C12
Relat. CPT2 Specific Activity (%)
Me-Lac
sgAars2 Scram #1 #2
d
Primary Myoblasts
WT
AARS2 OE
Aars2-/-
Relat. OCR (%)
Me-Lac
e
C2C12
α-KG Fraction (%)
M+5
M+4
M+3
M+2
M+1
M+0
Me-Lac
f
Alanine
β-Alanine
Lactate
PDB: 1YFS
W210
W161
D265
D217
V244
V196
N242
W194
G267
G219
M133
M92
g
Alanine
Lactate
β-Alanine
Dp (µcal/s)
Time (min)
ΔH (Kcal/mol)
Molar Ratio
N (sites)= 1.00
KD (M)= 1.49e-6±636e-9
ΔH (Kcal/mol)= -49.9±7.96
ΔG (Kcal/mol)= -7.87
-TΔS (Kcal/mol)= 42.0
N (sites)= 1.00
KD (M)= 1.64e-6±246e-9
ΔH (Kcal/mol)= -80
ΔG (Kcal/mol)= -7.76
-TΔS (Kcal/mol)= 72.2
N (sites)= 1.00
KD (M)= 2.14e-6±231e-9
ΔH (Kcal/mol)= -80
ΔG (Kcal/mol)= -7. 67
-TΔS (Kcal/mol)= 72.3
h
HEK293T
AARS2-Flag IgG
IP: PDHA1
Flag
PDHA1
Input
AARS2-Myc IgG
IP:Myc
Myc
i
HEK293T
AARS2-Myc
CPT2-Flag
IP:Flag
IP:Myc
70kDa

Supplementary information, Fig. S4 Lactate inactivated PDHA1 and CPT2 dependent on AARS2

a Me-Lac increases mitochondrial lactate levels. Mitochondrial lactate levels in C2C12 cells cultured in a medium supplemented with or without 2 mM and 10 mM Me-Lac, respectively (n=3), were measured. Success mitochondria extraction isolation was confirmed by staining both mitochondria maker COXIV and cytosolic marker GAPDH (here in after).

b-c Me-Lac inactivates cellular PDHA1 and CPT2 dependent on AARS2. Relative specific activities (to those of untreated C2C12 cells) of PDHA1 (**b**) and CPT2 (**c**) in C2C12 cells and *Aars2* knockout (KO) C2C12 cells that were untreated or treated with 10 mM Me-Lac (n=3) were determined.

d Me-Lac inactivates OCR dependent on AARS2 in mouse primary myoblasts. OCRs in wildtype, AARS2-overexpressing and *Aars2* knockout (KO) mouse primary myoblasts were determined (n=6).

e Lactate does not alter α-KG levels. Non-labeled (M+0) to all labeled (M+5) mitochondrial α-KG levels in C2C12 cells and 10 mM Me-Lac-treated C2C12 cells chased with 2 mM ^{13}C-glutamine for 1 h (n=3) were measured.

f Lactate and β-alanine may bind to the alanine-binding site of AARS2. Computer simulation showing that alanine (left), β-alanine (center), and lactate (right) may bind to the alanine-binding site of AARS2 in a similar configuration. Possible interactions are marked by dash lines.

g Lactate, alanine, and β-alanine bind to AARS2. The binding of 300 μM alanine (left), lactate (center), and β-alanine (right) to 50 μM recombinant AARS2 was assayed using ITC.

h, i AARS2 interacts with PDHA1 and CPT2. AARS2-PDHA1 interaction were detected between ectopically expressed AARS2 and endogenous PDHA1 (**h**), and AARS2-CPT2 interaction were detected between ectopically expressed AARS2 and CPT2 (**i**).

All data are reported as mean ± SEM of three independent experiments. Statistical significance was assessed by unpaired two-tailed Student's t-test and two-way ANOVA: *$P < 0.05$; **$P < 0.01$; ***$P < 0.001$; ****$P < 0.0001$; ns no significance.
