# Supplementary material for: Hypoxia induces mitochondrial protein lactylation to limit oxidative phosphorylation
Source: Cell Res. 2024 Jan 2;34(1):13–30. doi: 10.1038/s41422-023-00864-6 (PMC10770133; doi:10.1038/s41422-023-00864-6)
Supplement: Supplementary file 5 — Supplementary information, Fig. S5 [file 41422_2023_864_MOESM5_ESM.pdf]

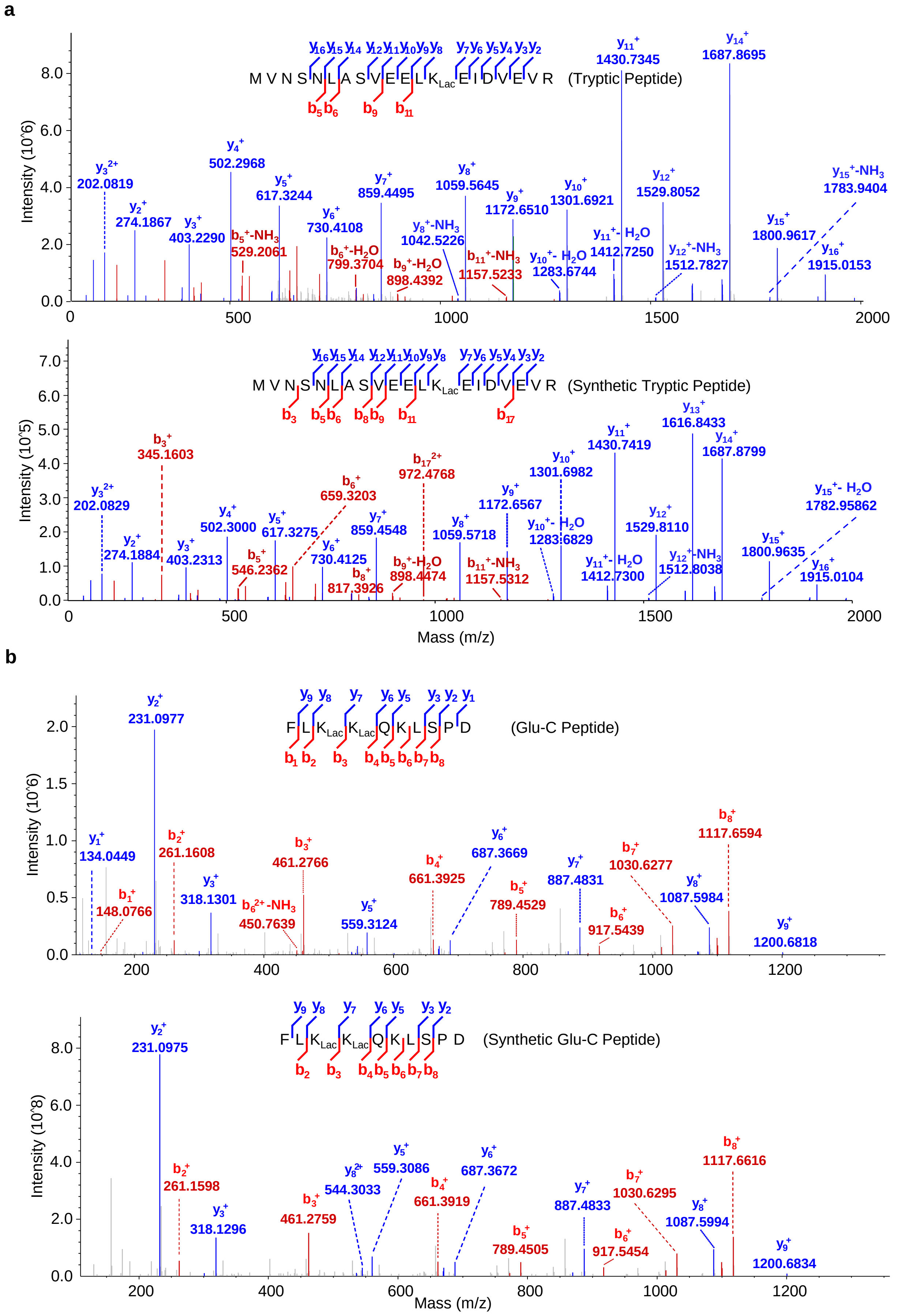
a
M V N S N L A S V E E L K Lac E I D V E V R (Tryptic Peptide)
Intensity (10^6)
M V N S N L A S V E E L K Lac E I D V E V R (Synthetic Tryptic Peptide)
Intensity (10^5)
Mass (m/z)
b
F L K Lac K Lac Q K L S P D (Glu-C Peptide)
Intensity (10^6)
F L K Lac K Lac Q K L S P D (Synthetic Glu-C Peptide)
Intensity (10^8)
Mass (m/z)

Supplementary information, Fig. S5 Identification of Lac-K336 in PDHA1 and Lac-K457/8 in CPT2

a, b The MS/MS spectra that led to the identification of Lac-K336 (**a**) and Lac-K457/8 (**b**) are shown. The MS/MS spectra identified via the PDHA1 tryptic peptides library (**a**, upper panel) aligned to the MS/MS spectra of synthetic Lac-K336-containing a PDHA1 tryptic peptide (**a**, lower panel), as well as MS/MS spectra identified via the CPT2 Glu-C peptides library (**b**, upper panel) aligned to the MS/MS spectra of synthetic Lac-K457/8-containing Glu-C peptide (**b**, lower panel) are shown.
