# Supplementary material for: Hypoxia induces mitochondrial protein lactylation to limit oxidative phosphorylation
Source: Cell Res. 2024 Jan 2;34(1):13–30. doi: 10.1038/s41422-023-00864-6 (PMC10770133; doi:10.1038/s41422-023-00864-6)
Supplement: Supplementary file 6 — Supplementary information, Fig. S6 [file 41422_2023_864_MOESM6_ESM.pdf]

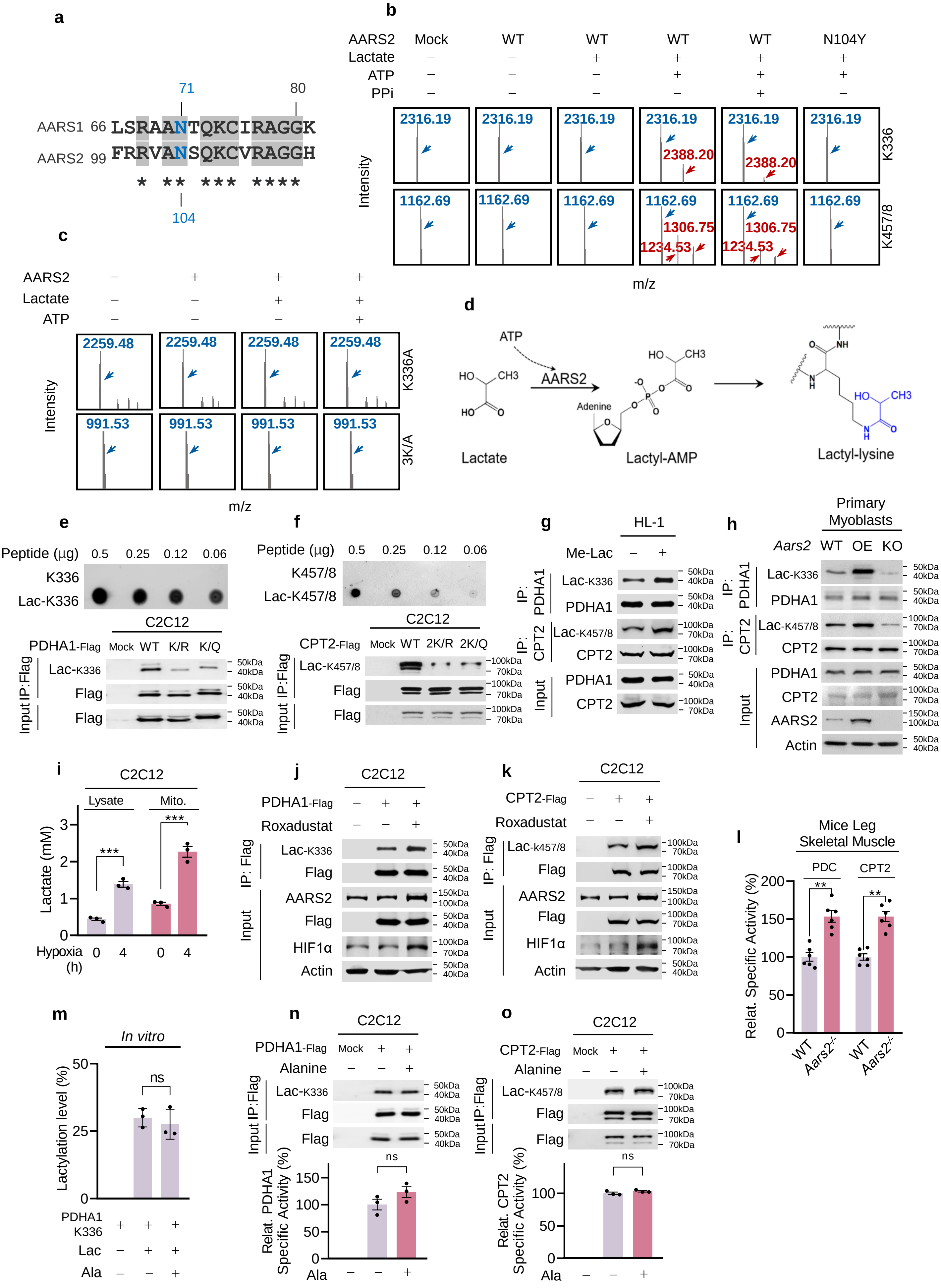

a
AARS1 66 LSRAANTQKCIRAGGK
AARS2 99 FRRVANSQKCVRAGGH
71
80
104
b
AARS2 Mock WT WT WT WT N104Y
Lactate
ATP
PPi
Intensity
m/z
K336
K457/8
2316.19
2388.20
1162.69
1306.75
1234.53
c
AARS2
Lactate
ATP
2259.48
991.53
K336A
3K/A
Intensity
m/z
d
ATP
AARS2
Lactate
Lactyl-AMP
Lactyl-lysine
Adenine
e
Peptide (µg) 0.5 0.25 0.12 0.06
K336
Lac-K336
C2C12
PDHA1-Flag Mock WT K/R K/Q
f
Peptide (µg) 0.5 0.25 0.12 0.06
K457/8
Lac-K457/8
C2C12
CPT2-Flag Mock WT 2K/R 2K/Q
g
HL-1
Me-Lac
h
Primary Myoblasts
Aars2 WT OE KO
i
C2C12
Lysate
Mito.
Lactate (mM)
Hypoxia (h)
j
C2C12
PDHA1-Flag
Roxadustat
k
C2C12
CPT2-Flag
Roxadustat
l
Mice Leg Skeletal Muscle
PDC
CPT2
Relat. Specific Activity (%)
m
In vitro
Lactylation level (%)
PDHA1 K336
Lac
Ala
n
C2C12
PDHA1-Flag
Alanine
Relat. PDHA1 Specific Activity (%)
o
C2C12
CPT2-Flag
Alanine
Relat. CPT2 Specific Activity (%)

Supplementary information, Fig. S6 Lactylation inactivates PDHA1 and CPT2

a AARS1 N71 corresponds to AARS2 N104. The conserved asparagine in AARS1 N71 and AARS2 N104 are aligned.

b AARS2 lactylates PDHA1 K336 and CPT2 K457/8 peptides *in vitro*. 10 mM lactate, 10 nM AARS2, and 0.05 mg/mL synthetic substrate peptide were included in the reaction mix. The reaction was determined at 37℃ for 3 h, which was subsequently desalted and subjected to analysis by mass spectrometer. MS results are shown (Fig. 5b).

c AARS2 fails to lactylate the peptides devoid of lysine. The ability of AARS2 to lactylate PDHA1 and CPT2 peptides in which K336 and K457/8 of each had been switched to alanine was detected by MS.

d Schematic of the catalytic mechanism underlying AARS2-mediated protein lysine lactylation. In the presence of AARS2, ATP and lactate are converted to lactyl-AMP, which contains a high-energy carbonyl-phosphate bond that modifies the ε-amine of lysine side chains.

e, f Verification of lactylation antibodies. Reactivities of site-specific PDHA1 K336 polyclonal antibody (**e**), and CPT2 K457/8 polyclonal antibody (**f**) were tested using dot blotting against synthetic unlactylated and lactylated peptides and western blotting to detect lactylation in PDHA1 and CPT2 and their lactylation-null mutants.

g Lactate-induced Lac-K336 and Lac-K457/8 in HL-1 cells. Lac-K336 levels and Lac-K457/8 levels of PDHA1 and CPT2, that was purified from HL-1 cells that had been cultured with or without 10 mM Me-Lac in the culture media, were detected.

h AARS2 regulates Lac-K336 and Lac-K457/8 in mouse primary myoblasts. Lac-K336 and Lac-K457/8 levels of PDHA1 and CPT2, that was purified from wildtype, AARS2-overexpressing and *Aars2* knockout (KO) mouse primary myoblasts, were detected.

i Hypoxia increases lactate levels in lysate and mitochondria. Lactate levels in lysate and in mitochondria of C2C12 cells that were cultured in a hypoxia chamber for indicated time durations, were detected (n=3).

j, k Roxadustat induces Lac-K336 and Lac-K457/8 in mouse leg skeletal muscles. Lac-K336 levels (**j**) and Lac-K457/8 levels (**k**) in PDHA1 and CPT2 purified from untreated and 50 μM roxadustat-treated C2C12 cells, were detected.

l AARS2 knockout (KO) activates PDC and CPT2. Specific activities of PDC and CPT2, isolated from WT and *Aars2* $^{-/-}$ mouse leg skeletal muscles were detected (n=6).

m Alanine does not affect lactylation of PDHA1 K336 *in vitro*. Physiologic levels (0.5 mM) alanine's ability to inhibit K336 lactylation *in vitro* was detected by mass spectrometer *in vitro* (n=3).

n, o Alanylation does not affect lactylation and the activities of PDHA1 and CPT2. The Lac-K336 levels and PDHA1 specific activity (**n**) as well as Lac-K457/8 levels and CPT2 specific activity (**o**) of ectopically expressed proteins in C2C12 cells and 10 mM alanine-treated C2C12 cells, were measured 4 h after treatment.

All data are reported as mean ± SEM of three independent experiments. Statistical significance was assessed by unpaired two-tailed Student's t-test and two-way ANOVA:***P* < 0.01; ****P* < 0.001; ns no significance.
