# Supplementary material for: Hypoxia induces mitochondrial protein lactylation to limit oxidative phosphorylation
Source: Cell Res. 2024 Jan 2;34(1):13–30. doi: 10.1038/s41422-023-00864-6 (PMC10770133; doi:10.1038/s41422-023-00864-6)
Supplement: Supplementary file 7 — Supplementary information, Fig. S7 [file 41422_2023_864_MOESM7_ESM.pdf]

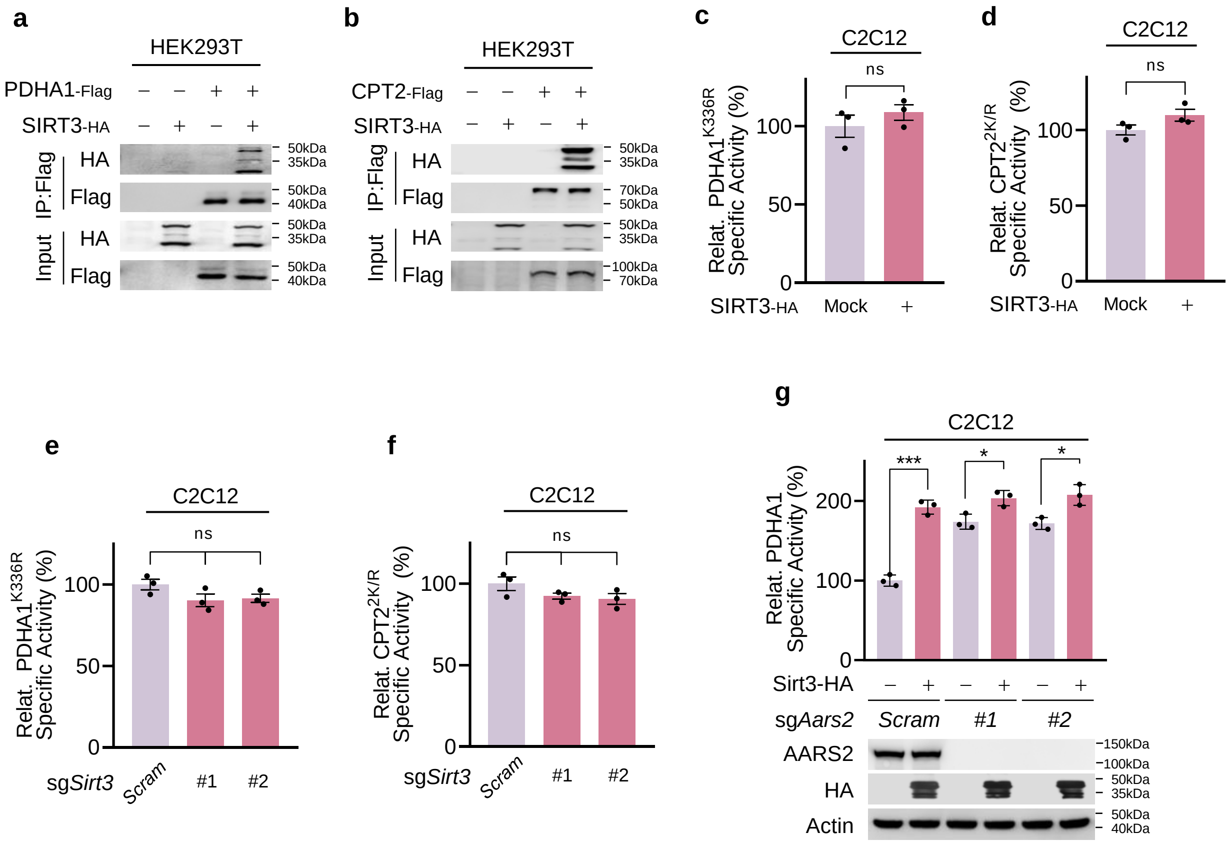
a
HEK293T
PDHA1-Flag
SIRT3-HA
IP:Flag
HA
Flag
Input
HA
Flag
50kDa
35kDa
50kDa
40kDa
50kDa
35kDa
50kDa
40kDa
b
HEK293T
CPT2-Flag
SIRT3-HA
IP:Flag
HA
Flag
Input
HA
Flag
50kDa
35kDa
70kDa
50kDa
50kDa
35kDa
100kDa
70kDa
c
C2C12
ns
Relat. PDHA1K336R Specific Activity (%)
100
50
0
SIRT3-HA
Mock
+
d
C2C12
ns
Relat. CPT22K/R Specific Activity (%)
100
50
0
SIRT3-HA
Mock
+
e
C2C12
ns
Relat. PDHA1K336R Specific Activity (%)
100
50
0
sgSirt3
Scram
#1
#2
f
C2C12
ns
Relat. CPT22K/R Specific Activity (%)
100
50
0
sgSirt3
Scram
#1
#2
g
C2C12
***
*
*
Relat. PDHA1 Specific Activity (%)
200
100
0
Sirt3-HA
sgAars2
Scram
#1
#2
AARS2
HA
Actin
150kDa
100kDa
50kDa
35kDa
50kDa
40kDa

Supplementary information, Fig. S7 SIRT3 regulates PDHA1 and CPT2 activities

a, b SIRT3 interacts with PDHA1 and CPT2. HA-tagged SIRT3 (result in two bands) was co-expressed with PDHA1 or CPT2 in HEK293T cells, following which co-immunoprecipitation between SIRT3 and PDHA1 (**a**) or CPT2 (**b**) was determined.

c-f SIRT3 does not exert an effect on the specific activities of lactylation site-null PDHA1 and CPT2. The specific activities of PDHA1 and CPT2 lactylation site mutants isolated from SIRT3-overexpressing (**c, d**) or *Sirt3* KO (**e, f**) C2C12 cells were compared (n=3).

g SIRT3 activates PDHA1 mainly through lactylation. The specific activities of PDHA1 isolated from C2C12 and *Aars2* knockout (KO) C2C12 cells that were transfected with empty vector or SIRT3 were determined (n=3).

All data are reported as mean ± SEM of three independent experiments. Statistical significance was assessed by unpaired two-tailed Student's t-test and two-way ANOVA: *$P < 0.05$; ***$P < 0.001$; ns no significance.
