# Supplementary material for: Hypoxia induces mitochondrial protein lactylation to limit oxidative phosphorylation
Source: Cell Res. 2024 Jan 2;34(1):13–30. doi: 10.1038/s41422-023-00864-6 (PMC10770133; doi:10.1038/s41422-023-00864-6)
Supplement: Supplementary file 8 — Supplementary information, Fig. S8 [file 41422_2023_864_MOESM8_ESM.pdf]

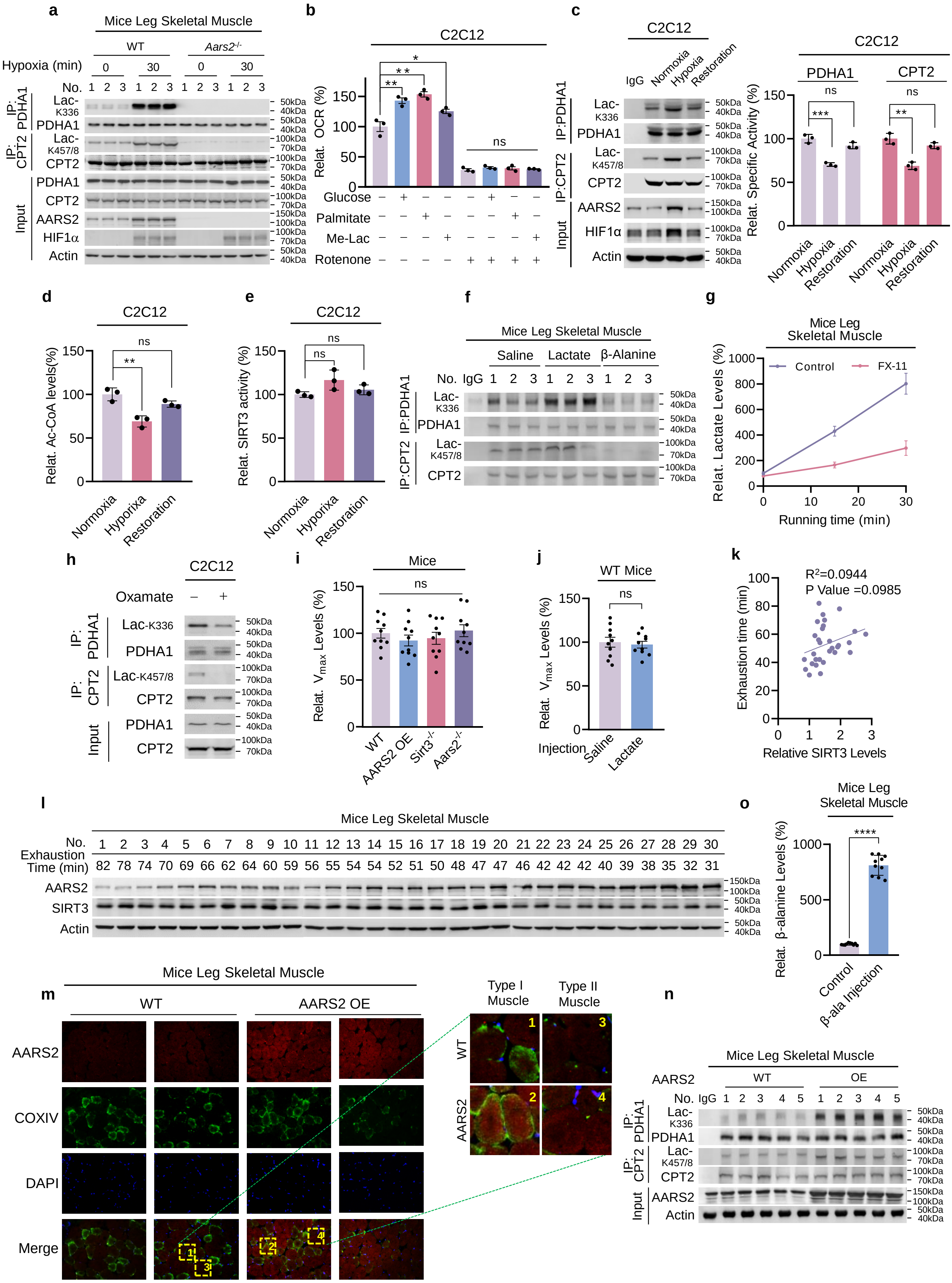

a
Mice Leg Skeletal Muscle
WT
Aars2-/-
Hypoxia (min)
0
30
No. 1 2 3 1 2 3 1 2 3 1 2 3
IP: PDHA1
Lac-K336
PDHA1
IP: CPT2
Lac-K457/8
CPT2
Input
PDHA1
CPT2
AARS2
HIF1α
Actin
50kDa
40kDa
100kDa
70kDa
150kDa
b
C2C12
Relat. OCR (%)
Glucose
Palmitate
Me-Lac
Rotenone
ns
c
C2C12
IgG
Normoxia
Hypoxia
Restoration
IP:PDHA1
IP:CPT2
Input
Lac-K336
PDHA1
Lac-K457/8
CPT2
AARS2
HIF1α
Actin
Relat. Specific Activity (%)
PDHA1
CPT2
d
C2C12
Relat. Ac-CoA levels(%)
Normoxia
Hyporixa
Restoration
e
C2C12
Relat. SIRT3 activity (%)
f
Mice Leg Skeletal Muscle
Saline
Lactate
β-Alanine
No. IgG 1 2 3 1 2 3 1 2 3
g
Mice Leg Skeletal Muscle
Relat. Lactate Levels (%)
Control
FX-11
Running time (min)
h
C2C12
Oxamate
i
Mice
Relat. V_{max} Levels (%)
WT
AARS2 OE
Sirt3-/-
Aars2-/-
j
WT Mice
Injection
Saline
Lactate
k
R^2=0.0944
P Value =0.0985
Exhaustion time (min)
Relative SIRT3 Levels
l
Mice Leg Skeletal Muscle
No. 1 2 3 4 5 6 7 8 9 10 11 12 13 14 15 16 17 18 19 20 21 22 23 24 25 26 27 28 29 30
Exhaustion Time (min) 82 78 74 70 69 66 62 64 60 59 56 55 54 54 52 51 50 48 47 47 46 42 42 42 40 39 38 35 32 31
AARS2
SIRT3
Actin
o
Mice Leg Skeletal Muscle
Relat. β-alanine Levels (%)
Control
β-ala Injection
m
Mice Leg Skeletal Muscle
WT
AARS2 OE
AARS2
COXIV
DAPI
Merge
Type I Muscle
Type II Muscle
n
Mice Leg Skeletal Muscle
AARS2
WT
OE
No. IgG 1 2 3 4 5 1 2 3 4 5

Supplementary information, Fig. S8 Lac-K336 and Lac-K457/8 regulate mice OCRs and endurance running abilities

a Hypoxia-induced AARS2 and Lac-K336 and Lac-K457/8 in mouse leg skeletal muscle. HIF1α, AARS2, Lac-K336 and Lac-K457/8 levels in dissected wildtype and *Aars2* KO mouse leg skeletal muscle perfused with Krebs Ringer solution aerated with or without O_2 in a 1200A Isolated Intact Muscle Test System for indicated time durations (n=3), were detected.

b Glucose, palmitate, and Me-Lac supplementation increases OCRs. OCRs in C2C12 cells cultured in DMEM base and glucose-, palmitate-, and Me-lac-supplemented DMEM media were tested under the presence or absence of 1 μM rotenone.

c-e Reoxygenation restored CPT2 and PDHA1 lactylation and activities. Lactylation and activities of CPT2 and PDHA1 (**c**), Ac-CoA levels (**d**) and activities of SIRT3 (**e**) were detected in C2C12 cells under normoxia, hypoxia and 30 min after reoxygenation (n=3).

f Lactate enhances Lac-K336 and Lac-K457/8 in mice, while β-alanine inhibits them. Leg muscle Lac-K336 and Lac-K457/8 levels in 2 g/kg saline-, lactate-, or β-alanine-leg injected mouse leg gastrocnemius muscles (n=3) were detected.

g FX-11 reduces lactate production in mouse leg skeletal muscle during running. Lactate levels in the leg skeletal muscles of untreated mice and mice treated with 2 mg/kg FX-11 (intraperitoneally injected for 2 weeks) were measured 0, 15, and 30 min after running (n=6).

h Oxamate decreases Lac-K336 and Lac-K457/8. Lac-K336 and Lac-K457/8 were detected in C2C12 cells treated or untreated with 10 mM oxamate for 24 h.

i, j Lactylation exerts only a limited effect on mouse type II fast twitch muscle. Sprint abilities of wildtype, AARS2-overexpressing, *Sirt3*$^{-/-}$ and *Aars2*$^{-/-}$ mice (**i**, n=10) and wildtype mice intramuscularly injected with saline or 2 g/kg lactate (**j,** n=10) were compared.

k Running exhaustion time is moderately correlated with mouse leg skeletal muscle and SIRT3 background. The correlation between mouse running exhaustion time and mouse leg skeletal muscle SIRT3 levels was analyzed (n=30; Supplementary information, Fig. S8l for western blot data).

l SIRT3 and AARS2 levels and the running exhaustion times of mice. The SIRT3 and AARS2 levels of mouse leg skeletal muscles, detected via western blot, as well as running exhaustion times were recorded for each mouse (n=30, Fig. 7g, Supplementary information, Fig. S8k).

m Muscle-specific overexpression of AARS2. AARS2 in mouse leg skeletal muscles was stained using AARS2 antibody (red), and mitochondria (COXIV staining, green) were employed to distinguish between type I and type II muscles. Enlarged views (1-4) showing AARS2 overexpression in both type I and type II muscles are displayed on the right (n=2).

n AARS2 overexpression increases Lac-K336 and Lac-K457/8 levels. The Lac-K336 and Lac-K457/8 levels of wildtype and muscle-specific AARS2-overexpressing mouse leg skeletal muscles were detected (n=5).

o β-alanine injection increases leg muscle β-alanine levels. β-alanine levels in leg muscles were detected in untreated and 2 g/kg β-alanine- leg injected mouse leg gastrocnemius muscles (n=10).

All data are reported as mean ± SEM of three independent experiments. Statistical significance was assessed by unpaired two-tailed Student's t-test and two-way ANOVA: **P* < 0.05; ***P* < 0.01; ****P* < 0.001; *****P* < 0.0001; ns no significance.
