# Supplementary material for: Hypoxia induces mitochondrial protein lactylation to limit oxidative phosphorylation
Source: Cell Res. 2024 Jan 2;34(1):13–30. doi: 10.1038/s41422-023-00864-6 (PMC10770133; doi:10.1038/s41422-023-00864-6)
Supplement: Supplementary file 9 — Supplementary information, Fig. S9 [file 41422_2023_864_MOESM9_ESM.pdf]

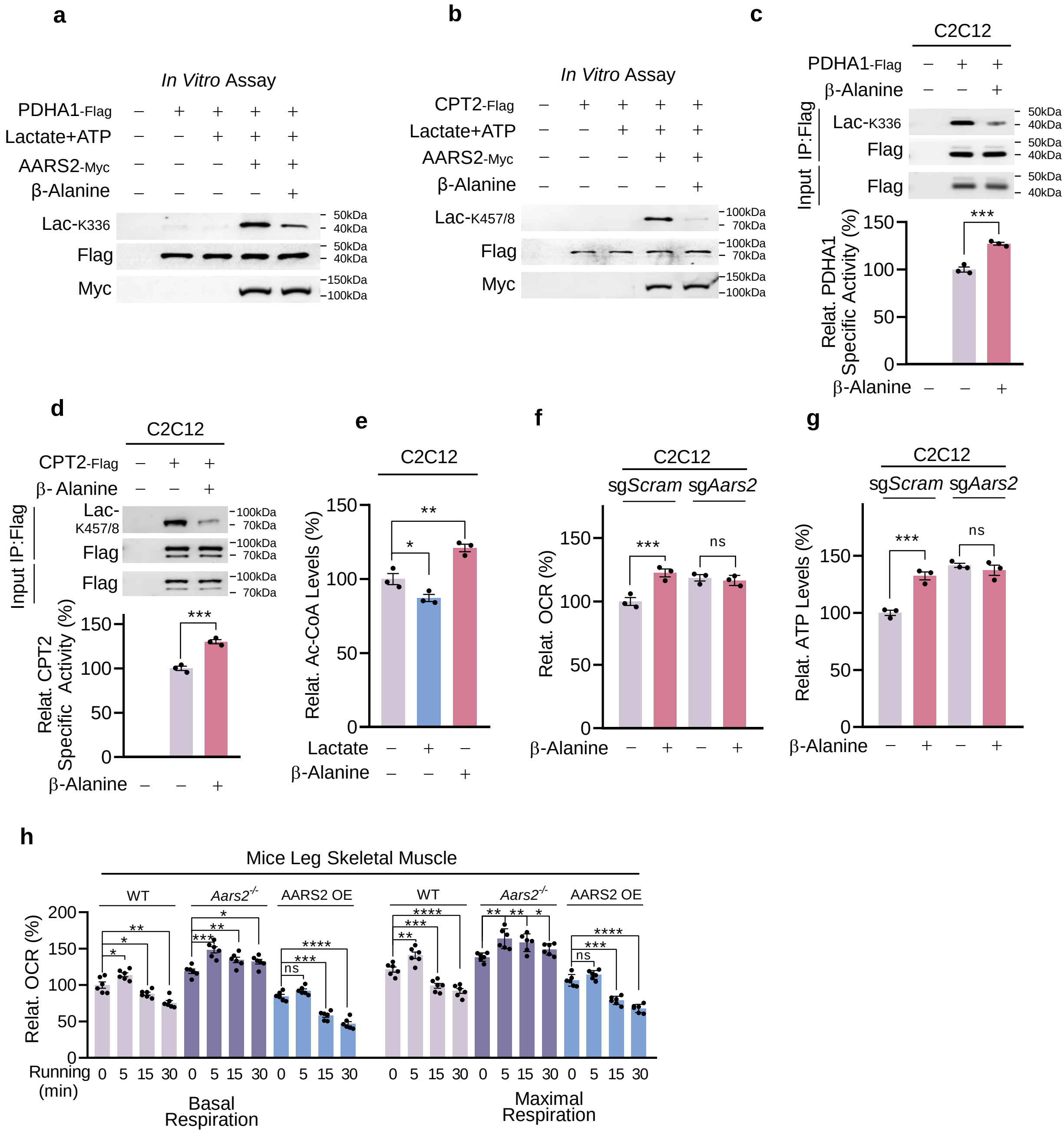
a
In Vitro Assay
PDHA1-Flag
Lactate+ATP
AARS2-Myc
β-Alanine
Lac-K336
Flag
Myc
50kDa
40kDa
150kDa
100kDa
b
CPT2-Flag
Lac-K457/8
70kDa
c
C2C12
Input IP:Flag
Relat. PDHA1 Specific Activity (%)
d
Relat. CPT2 Specific Activity (%)
e
Relat. Ac-CoA Levels (%)
Lactate
f
sgScram sgAars2
Relat. OCR (%)
ns
g
Relat. ATP Levels (%)
h
Mice Leg Skeletal Muscle
WT
Aars2-/-
AARS2 OE
Running (min)
Basal Respiration
Maximal Respiration

Supplementary information, Fig. S9 β-alanine regulates Lac-K336, Lac-K457/8, and respiration

a, b β-alanine inhibits AARS2 lactyltransferase *in vitro*. The ability of recombinant AARS2 to catalyze Lac-K336 in recombinant PDHA1 (**a**) and Lac-K457/8 in recombinant CPT2 (**b**) was analyzed in the absence or presence of β-alanine.

c, d β-alanine activates PDHA1 and CPT2. The levels of Lac-K336 and Lac-K457/8 and specific activities of ectopically expressed PDHA1 (**c**) and CPT2 (**d**) isolated from C2C12 or 5 mM β-alanine-treated C2C12 cells (n=3), were compared.

e The effects of β-alanine on Ac-CoA levels are the opposite of that shown by lactate. The cellular Ac-CoA levels of C2C12, and C2C12, treated with either 10 mM lactate or 5 mM β-alanine (n=3), were compared.

f, g β-alanine regulates OCRs and ATP levels in an AARS2-dependent manner. The OCR (**f**) and ATP (**g**) levels of C2C12 and *Aars2* KO C2C12 cells with or without β-alanine treatment were detected (n=3).

h Lactylation inhibits basal and maximal respiration in mouse leg skeletal muscle. Basal and maximal respiration in WT, *Aars2*$^{-/-}$ and AARS2 OE mouse leg skeletal muscle sampled after running for indicated times (n=6) were detected.

All data are reported as mean ± SEM of three independent experiments. Statistical significance was assessed by unpaired two-tailed Student's t-test and two-way ANOVA: *$P < 0.05$; **$P < 0.01$; ***$P < 0.001$; ****$P < 0.0001$; ns no significance.
