# Supplementary material for: Hypoxia induces mitochondrial protein lactylation to limit oxidative phosphorylation
Source: Cell Res. 2024 Jan 2;34(1):13–30. doi: 10.1038/s41422-023-00864-6 (PMC10770133; doi:10.1038/s41422-023-00864-6)
Supplement: Supplementary file 10 — Supplementary information, Table. S1 [file 41422_2023_864_MOESM10_ESM.pdf]

Supplementary information, Table. S1 Original proteomic data of MS results.

Original proteomic data can be obtained from https://www.ebi.ac.uk/pride/

Project Name	RRIDE Accession number
Synthetic hydroxylated Alanyl-tRNA synthetase 2 Glu-C peptide	PXD037456
Hydroxylation of alanyl-tRNA synthetase 2 Glu-C peptide	PXD037463
Synthetic lactylated pyruvate dehydrogenase E1 component subunit alpha tryptic peptide	PXD037457
Lactylation of pyruvate dehydrogenase E1 component subunit alpha tryptic peptide	PXD037462
Synthetic lactylated carnitine palmitoyltransferase 2 Glu-C peptide	PXD037458
Lactylation of carnitine palmitoyltransferase 2 Glu-C peptide	PXD037461
